# Genetic depletion of the early autophagy protein ATG13 impairs mitochondrial energy metabolism, augments oxidative stress, induces the polarization of macrophages to the M1 inflammatory mode, and compromises myelin integrity in skeletal muscle

**DOI:** 10.1007/s00011-025-02158-6

**Published:** 2026-01-27

**Authors:** Mubaraq A Toriola, Emma Timlin, Sarojini Bulbule, Amy Reyes, Omolola Mary Adedeji, C Gunnar Gottschalk, Animesh Barua, Leggy A Arnold, Avik Roy

**Affiliations:** 1 Milwaukee Institute for Drug Discovery, University of Wisconsin- Milwaukee, 2000 E Kenwood Blvd, Milwaukee, WI 53211 USA; 2 Department of Chemistry & Biochemistry, University of Wisconsin-Milwaukee, 2000 E Kenwood Blvd, Milwaukee, WI 53211 USA; 3 Simmaron Research R&D laboratory, 2000 E Kenwood Blvd, Suite#320, Milwaukee, WI 53211 USA; 4 Simmaron Research INC, 948 Incline Village, Milwaukee, NV 89451 USA; 5 Milwaukee Institute for Drug Discovery, University of Wisconsin-Milwaukee Chief Scientific Officer Simmaron Research Institute Research and Development laboratories, 2000 E Kenwood Blvd Suites #320, Milwaukee, WI 53211 USA

## Abstract

**Objective:**

M1 macrophage activation is crucial in chronic inflammatory diseases, yet its molecular mechanism is unclear.

**Results:**

Our study showed that hemizygous deletion of the early autophagy gene atg13 (Tg+/-ATG13) disrupts cellular autophagy, hinders mitochondrial oxidative metabolism, and increases reactive oxygen species (ROS) levels in splenic macrophages, leading to M1 polarization. After reducing the expression of the autophagy markers WDFY3 and LC3, flow cytometric analysis of M1/M2 markers (CD40, CD86, CD115, CD163, and CD206), decreasing oxygen metabolism, as evaluated by the ROS-sensor dye DCFDA, and Seahorse oxygen consumption studies revealed that ablation of the atg13 gene impairs mitochondrial function, triggering M1 polarization. Additionally, redox imbalance may impair Sirtuin-1 activity via nitrosylation, increasing the level of acetylated p65 in macrophages and contributing to the inflammatory response in M1Mφs. Additionally, ablation of the atg13 gene resulted in increased infiltration of M1Mφs into the muscle vasculature, deterioration of myelin integrity in nerve bundles, and a reduction in muscle strength following treadmill exercise.

**Conclusions:**

Our study shows that impaired ATG13-driven autophagy increases inflammation through sirtuin-1 inactivation and NF-κB activation, suggesting a role for ATG13 in post-exertional malaise (PEM).

**Supplementary Information:**

The online version contains supplementary material available at 10.1007/s00011-025-02158-6.

## Highlights

1. Genetic deletion of the atg13 gene induced the polarization and activation of M1Mφ cells in the spleen.

2. The suppression of ATG13 induced mitochondrial deficits in oxygen consumption and augmented ROS production in splenic M1Mφ cells.

3. A deficit in oxygen metabolism in mitochondria inhibited the cellular acetylase SIRT1 via nitrosination and triggered inflammation via acetylation of p65.

4. The active infiltration of M1Mφ cells in the muscle vasculature contributed to the loss of myelin integrity in muscle-serving nerves.

**Supplementary Information:**

The online version contains supplementary material available at 10.1007/s00011-025-02158-6.

## Introduction

 Autophagy [1] is a mechanism of cellular quality control metabolism in which defective cellular proteins [2] and depolarized mitochondria [3] are enclosed in a bilayer vesicle known as the autophagosome, which is subsequently targeted and fused to the lysosome for degradation [4]. The formation of the autophagosome [5] is programmed by the concerted action of a class of proteins termed autophagy-related proteins or ATGs [6]. Over 36 ATG proteins [7], primarily identified from yeast autophagy mutants [8], drive the autophagic process [9]. ATG13 primarily regulates autophagosome formation in the context of starvation-induced conditions [10]; however, it also plays a critical role in starvation-independent or constitutive autophagy [11]. The ATG9/2/18 transmembrane complex promotes phagophore expansion by shuttling the membrane from sources such as the endoplasmic reticulum, trans-Golgi network, and mitochondria [12]. The ATG7/ATG3/Atg8 and ATG7/ATG10/ATG12 ubiquitin-like conjugation systems are involved in enclosing autophagosomal membranes [13].

The involvement of ATG13 in both health and disease is increasingly being elucidated. Loss of autophagy resulting from the genetic ablation of atg13 has been demonstrated to suppress cardiac development [14, 15], indicating that ATG13 may be essential for maintaining cardiac function [16]. Recent studies have additionally revealed an autophagy-independent role for ATG13, supporting its contribution to the suppression of viral gene expression [17] and conferral of protection against viral infections [18]. This specific mechanism of ATG13 appears relevant to both limiting chronic viral infections and preventing viral reactivation. Historically, impairments in these functions may contribute to persistent pathological conditions, such as post-infectious fatigue. Therefore, the role of ATG13 in regulating innate immunity needs to be studied thoroughly. Our previous studies [19, 20] demonstrated that the loss of function of the atg13 gene in a Tg-ATG13 knockout mouse model impaired autophagy, which led to the increased infiltration of inflamed mononuclear cells into the vasculature of the muscle parenchyma, which potentially caused a demyelinating response in muscle-serving nerves, resulting in chronic muscle fatigue. Further analysis revealed that these mononuclear cells were CD40^+^ and iNOS^+^ inflammatory macrophage (Mφ) cells. However, the mechanism by which *atg13* gene ablation triggers inflammation in Mφ cells is not known.

The inflammatory and chronic demyelinating pathologies resulting from *atg13* ablation may be pertinent to the pathogenesis of neuroinflammatory diseases. In this study, we emphasize the impact of *atg13* deficiency in inducing inflammatory and demyelinating alterations within nerve fibers that serve skeletal muscle tissue. Myalgic encephalomyelitis or chronic fatigue syndrome (ME/CFS) [20] is characterized as a persistent inflammatory condition marked by diminished muscle strength, which worsens following physical or cognitive exertion—a phenomenon commonly referred to as postexertional malaise (PEM). Accordingly, this research may provide valuable insights into the molecular mechanisms underlying PEM. PEM is a key symptom of ME/CFS syndrome [21, 22] that involves significant muscle fatigue, pain, and cognitive deficits following physical or emotional exertion. Recent studies have indicated that glucose metabolism deficits [23], reduced mitochondrial oxygen consumption [24], defective energy metabolism [25], and immune cell depletion [26] may contribute to PEM, although the underlying molecular mechanism remains unclear. In our current study, comprehensive flow cytometry analyses revealed that Tg^+/−ATG13^ mice with significant depletion of the atg13 gene exhibited M1Mφ polarization in the spleen. Mitochondrial stress studies, such as oxygen consumption analyses and ROS production studies, further indicated that these Tg^+/−ATG13^ M1Mφ cells are deficient in oxygen consumption and energy metabolism, which may trigger an inflammatory response, causing enhanced infiltration in the muscle vasculature and potential demyelination in muscle-serving nerves. Overall, our present study delineates the molecular role of the atg13 gene in promoting muscle inflammation via the activation of M1Mφ cells.

## Materials and methods

### The following reagents, media, buffers, kits, and antibodies were used

TBS (10×) (Ref#28358), 20X PBS-Tween (Ref#28352), NuPAGE™ MOPS SDS Running Buffer (Ref#NP0001), 20X NuPAGE™ Transfer Buffer (Ref# NP00061), 20X TE Buffer (Cat#TE03), and 5X TBE (Cat#J63487)., NuPAGE™ 4–12% Bis-Tris Gel (Ref# NP0322BOX), and nitrocellulose membrane (0.45 μm; Ref# 88025) were purchased from ThermoFisher Scientific (MA, USA). Bafilomycin A1 (Cat# sc-201550 A)was purchased from Santa Cruz Biotechnology, INC. SBI-0206965 (Cat#SML1540) was purchased from Millipore Sigma. DMEM (Cat # 11995-065) and STEMpro^®^ Accutase^®^ (Ref# A11105-01) were purchased from Gibco (ThermoFisher Scientific). Intercept TBS blocking buffer (Part# 927-60001), antibody diluent (Part# 9297–95001), IRDye™ 680 donkey anti-rabbit (Part# 926-68073), IRDye™ 680 donkey anti-mouse (Part# 926-68072), IRDye™ 680 goat anti-rabbit (Part# 926-68071), IRDye™ 680 goat anti-mouse (Part# 926-68070), and nitrocellulose membrane (Part# 926-31090) were purchased from LICOR Biosciences (NE, USA). Triton X (0.05%)-conjugated eBioscience™ Flow Cytometry Staining Buffer (Ref# 00-4222-57; ThermoFisher, Inc.) was used to wash, neutralize nonspecific binding and achieve optimum permeabilization for intracellular staining. All the primary antibodies used are listed below, with the catalog number, vendor, and application table 1.

**Table 1:** The list of antibodies and their application

Antibody (conjugate)	Catalog#	Vendor	Application (dil^*n*^)
CD40	PA5-78980	Invitrogen	IB (1:500), IF (1:100)
LAMA5	PA5-49930	Invitrogen	IF (1:100)
ATG13	10181-2-AP	Protein tech	IB (1:500), IF (1:100)
ATG101	PA5-114272	Invitrogen	IB (1:200)
Beta-actin	PA5-85271/AM4302	Invitrogen	IB (1:1000)
IBA1	MA5-50413	Invitrogen	IF (1:500)
S468P p65	500-11854	Amsbio	IF (1:250)
Rabbit LC3	14600-1-AP	Protein tech	IB, IF (1:250)
WDFY3	55009-1-AP	Protein tech	IHC (1:100), FACS (1:100)
3Nitrotyrosine	MA1-12770	Invitrogen	IF (1:100)
Acetyl p65	PA5-17264	Invitrogen	IF(1:100), IB (1:250)
SIRT1	MA5-30879	Invitrogen	IF (1:100); IB (1:250)
SIRT2	PA5-20487	Invitrogen	IB (1:500)
iNOS	18985-1-AP	Invitrogen	IF (1:100)
CD163(APC)	MA5-17717	Invitrogen	FACS (1:100)
CD11b (FITC)	11-0118-42	Invitrogen	FACS (1:100)
CD40 (APC)	17-0409-42	Invitrogen	FACS (1:100)
CD86 (PE)	12-0869-42	Invitrogen	FACS (1:100)
Arginase1 (APC)	17-3697-82	Invitrogen	FACS (1:100)
CD206 (APC)	17-2069-42	Invitrogen	FACS (1:100)
P62 (SQSTM1)	18420-1-AP	ProteinTech	IB (1:250), IF (1:100)

*Generation of Tg*^*+/− ATG13*^ *mice and phenotype assessment* : The ATG13 repressor mouse strain used for this research project, C57BL/6 N-Atm1Brd Atg13tm2a (EUCOMM)Hmgu/BayMmucd, RRID: MMRRC_041527-UCD, was obtained from the Mutant Mouse Resource and Research Center (MMRRC) at the University of California at Davis, an NIH-funded strain repository, and was donated to the MMRRC by Arthur Beaudet, M.D., Baylor College of Medicine. Mice were generated at the Baylor College of Medicine as part of the Baylor College of Medicine, Sanger Institute, and MRC Harwell (BaSH) Consortium for the National Institutes of Health (NIH) Common Fund program for Knockout Mouse Production and Cryopreservation (1U42RR033192-01) and Knockout Mouse Phenotyping (1U54HG006348-01). After generation of the strain, the ATG13 repressor mice were transported, housed, and bred at the CRC animal facility of the UWM according to the approved IACUC protocol 22–23 #20. To selectively knock out the atg13 gene, these mice were bred with B6. C-Tg(CMV-cre)1Cgn/J, a mouse that expresses the Cre recombinase enzyme and was designed to preferentially delete the loxP-flanked *atg13* gene in males (via X-linked gene transmission). After 5 generations of breeding and genotyping, we were unable to obtain any homozygous *atg13* knockout offspring, possibly due to increased lethality during the gestational period. However, after breeding for 7 generations, we successfully generated a sufficient number of hemizygous male mice. Although there was no apparent developmental deficit or growth disorder, these mice displayed slower movement, anxiety, and poor motor performance once subjected to treadmill stressors. Male mice gain body weight faster than wild-type mice start at 6 months of age, and their abdominal fat content significantly increases. The following primer pairs (postCre: product length = 797 bp) were used for identifying the Tg^+/ATG13^ genotype. Primer#1: 5’---GCTACCATTACCAGTTGGTCTGGTGTC—3’ and Primer #5: 5’---CACCATCTGTAATGGGATCCAAAGGC—3’. The preCre band appears at 499 bp.

### Preparation of splenocytes and enrichment of macrophages (Mφs)

To isolate Mφ-enriched splenocytes, spleen tissue was harvested and homogenized in 5 mL of sterile HBSS for 2 min. The mixture was centrifuged at 1200 rpm for 5 min and then digested with 1 mL of accutase enzyme at 37 °C for 5 min. The digestion mixture was neutralized with 1X HBSS, followed by centrifugation and resuspension in complete DMEM/F12 media. The pellet was disintegrated by pipetting and seeded in a flask at 37 °C for 10 min, after which the media containing the suspended cells was removed. The adherent cells were primarily Mφs with some monocytes. The cells were carefully harvested in serum-free DMEM/F12 for subsequent immunoassays without any further addition of M1- or M2-specific growth factors. This condition was essential for our study to minimize potential confounding variables arising from media composition.

**Fig. 1:**
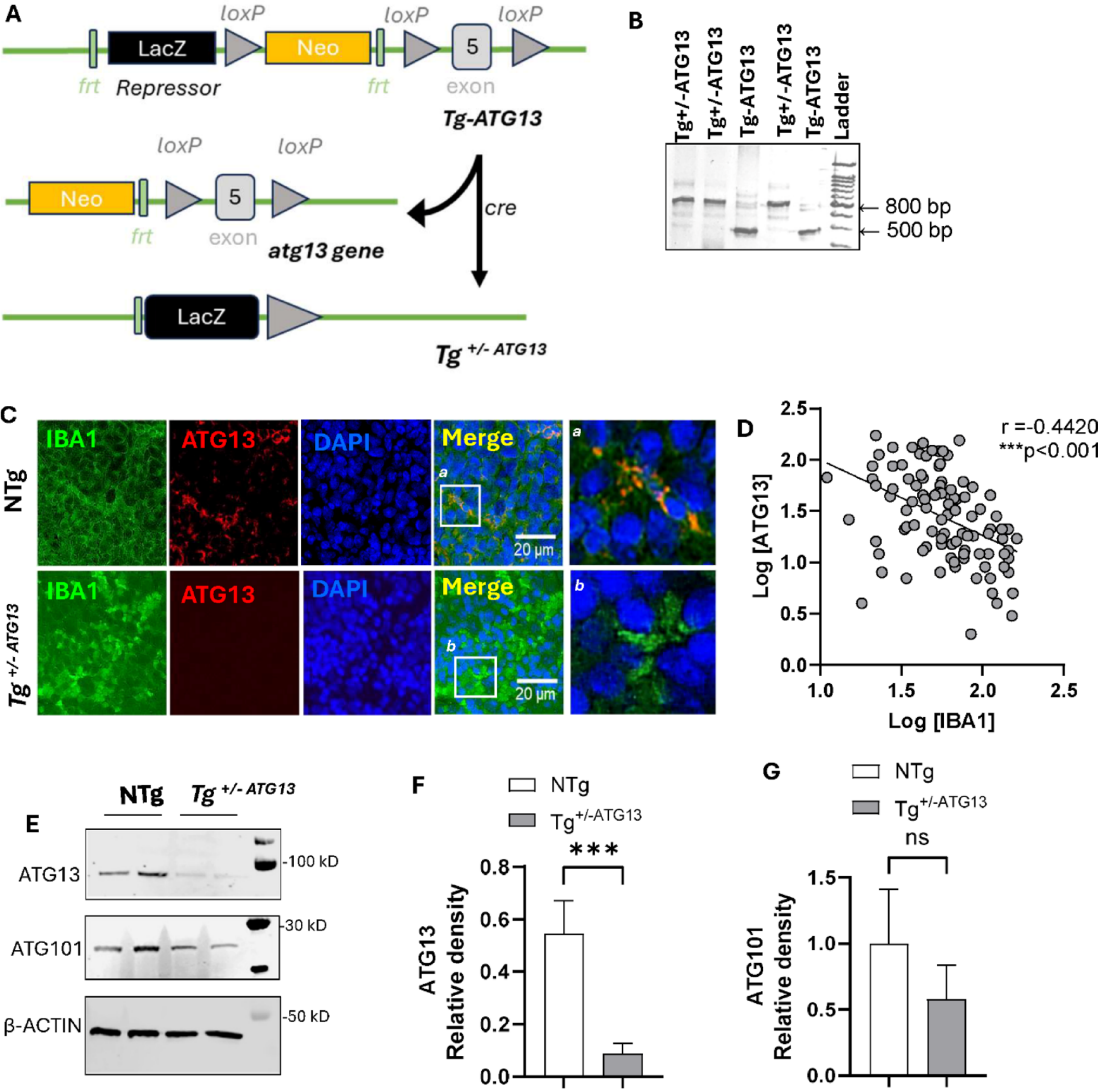
Generation of Tg^+/-ATG13^ mice and evaluation of ATG13 expression in the spleen. **A** A schematic strategy for the generation of Tg^+/-ATG13^ mice. **B** Genotype data generated with different primer sets (as mentioned in the Methods section) for identifying the LacZ repressor Tg-ATG13 (~ 500 bp = 450 bp) and Tg^+/-ATG13^ (~ 800 bp = 797 bp) mice. Nontransgenic (NTg) mice did not show any band at ~ 500 or ~ 800 bp. **C** Splenic tissue from 10-week-old male NTg and Tg^+/-ATG13^ mice (*n* = 5/group) was paraffin-embedded, cut at 5 μm thickness, and double immunostained for ATG13 and the activated Mφ marker IBA1. **D** The parametric Pearson correlation was performed to monitor the relationship between IBA1 and ATG13 expression in 100 dual-stained cells. A linear regression plot was generated in GraphPad Prism 10, which indicated that the slope (Pearson coefficient = *r* = -0.4420) was significantly nonzero, with F_1,98_ =23.80 and **p < 0.001. **E** Immunoblot analyses of ATG13, ATG101, and the loading control β-actin are shown . The relative densities of **F** ATG13 and **G** ATG101 were calculated as the ratio of the target band intensity to the respective actin band density and are plotted as histograms. The Mann‒Whitney U test was performed to test the significance of the difference in the means between groups; ***p < 0.001 vs. ATG13 of NTg. NS = nonsignificant. The results were confirmed after three independent experiments were performed for *n* = 5 animals/group

### Flow cytometry analysis

Enriched Mφs were isolated from the spleens of 10- to 12-week-old male NTg and Tg ^+/− ATG13^ mice as described above, after which the cells were counted with a CountessTM 3 automated cell counter (ThermoFisher Scientific, MA, USA). A BD Accuri™ C6 Plus (BD Biosciences, San Jose, CA) flow cytometer, which was equipped with a blue (488 nm) and red (640 nm) laser, two light scatter detectors and four fluorescence detectors with optical filters (FL1 = 533/30, FL2 = 585/40, FL3 = 670 LP/610/20 and FL4 = 675/25), was used. At least 10,000 total events were calculated for the analyses once compensation was performed using the CompBead Fluorescence matrix according to the manufacturer’s recommendations. Briefly, cells were suspended in eBioscience™ Flow Cytometry Staining Buffer (Ref# 00-4222-57; ThermoFisher, Inc.), stained with FITC-, PE-, or APC-conjugated antibodies for 30 min at room temperature, washed (3X), and then subjected to analysis via a flow cytometer. The results were analyzed using FlowJo V10.10.0 software as per standard guidelines.

### Immunofluorescence (IF) analysis:

 IF analysis was performed as described elsewhere [27]. Muscle tissue was mounted in paraffin-embedded blocks and then sectioned at a thickness of four microns with our Leitz 1512 manual microtome. Subsequently, the sections were sequentially treated with xylene; 100%, 70%, 50%, and 20% ethanol; and rehydrated in water following the standard IHC protocol. Blocking was performed using 2% BSA buffer, followed by overnight incubation with primary antibodies at room temperature. The tissue sections were washed with 1X PBST and then incubated with a biotinylated secondary antibody. For DAB staining, biotin-conjugated 2^◦^ antibodies were used per the instructions of the dual IHC kit (Abcam; Cat # ab210059). For immunofluorescence, FITC- and TRITC-conjugated secondary antibodies (Jackson ImmunoResearch) were used. The slides were deparaffinized, subjected to antigen retrieval in citrate buffer (pH 5.5), blocked with 2% horse serum, treated with primary antibodies (1:100–1:250), washed with 1× TBST, incubated with labeled antibodies, washed again (with DAPI included in the final wash at a dilution of 1:10,000), and cover slipped. Imaging was performed using an Accu-scope fluorescence microscope.

The mean fluorescence intensity (MFI) was measured using Fiji-ImageJ software. The process involved opening the image from the “File” menu and then separating the channels via the “Color” option in the “Image” menu. The green or red channel images were chosen for MFI measurement by selecting “Mean gray value” under “Set Measurements” in the “Analyze” menu. The polygon tool was applied to define the region of interest, and the MFI was obtained by pressing “Ctrl + M” on the keyboard.

To visualize myelin integrity and exposed axons, a surface plot was drawn by using the “interactive 3D surface plot version 3.24” module in ImageJ software.

Cell counting was conducted with CaptaVision + software (Accu-Scope, Inc.). The image was opened and calibrated to 6 pixels/µm according to the raw image resolution. Using the “Measure” tool, the “Manual Class counting” function was used, and cells were counted by clicking on each target cell.

**Fig. 2:**
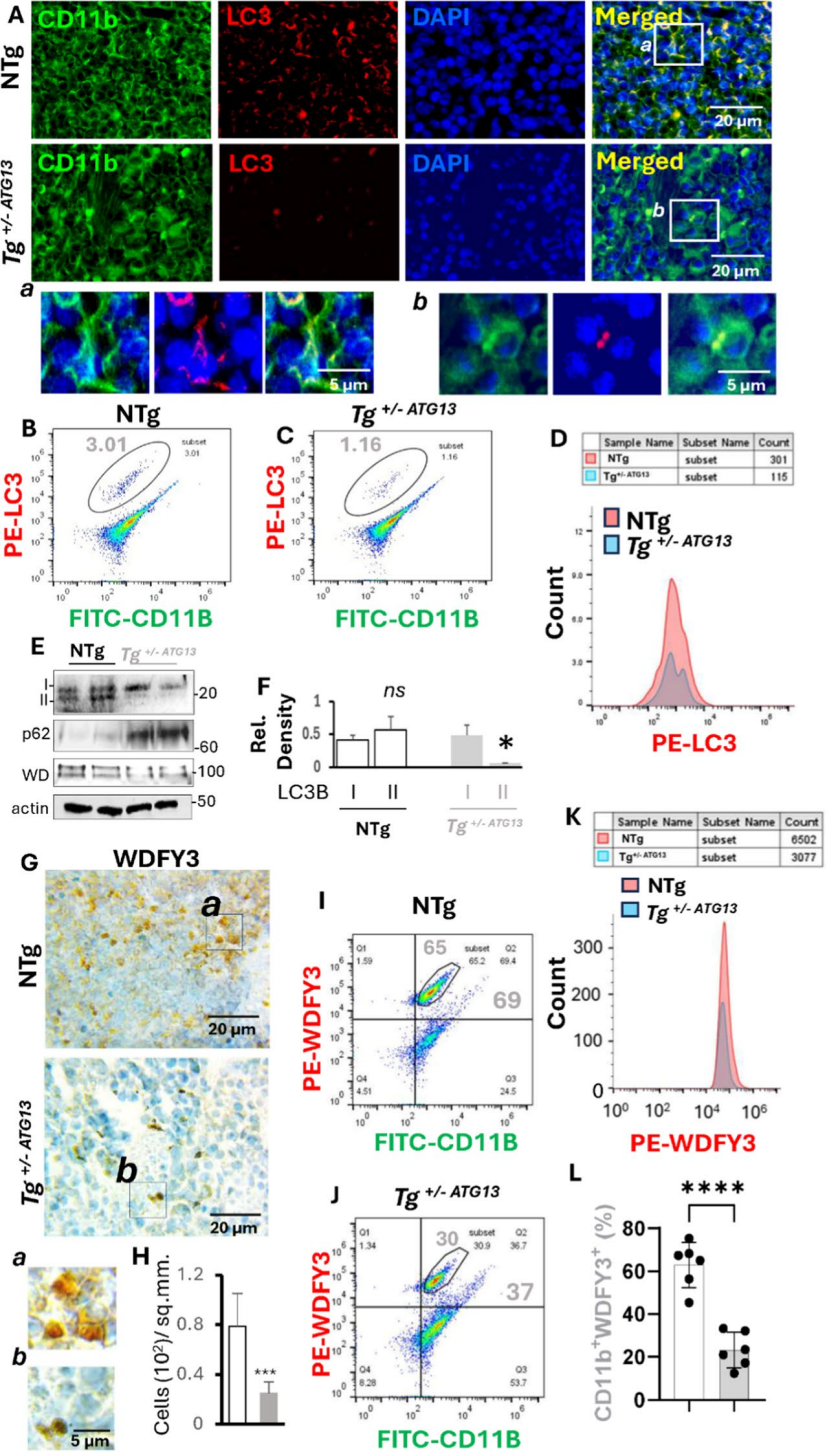
Autophagy impairment in the splenic Mφs of Tg^+/-ATG13^ mice. **A** Dual IF analysis of LC3 (rabbit anti-LC3; Cat#; ProteinTech; dilution 1:100) and CD11b (mouse anti-CD11b; Cat#; Invitrogen; dilution 1:100) in 5 μm thick paraffin-embedded sections of 10–12-week-old nontransgenic and hemizygous atg13 knockout mice (*n* = 6/group). (*Inset*) Magnified dual IF images of (a) NTg and (b) Tg^+/-ATG13^. Dual flow cytometry of PE-labeled LC3 and FITC-labeled CD11b in purified Mφs isolated from 10- to 12-week-old **B** NTg and **C** Tg^+/- ATG13^ mice (male). The total number of gated events was 20,000/group. The cells under the enclosed area represent a distinct population of CD11b-ir cells, which are also LC3-positive. **D** Histogram analyses to quantify the CD11b^+^LC3^+^ cells among the NTg and Tg^+/-ATG13^ Mφs. **E** Immunoblot analyses of LC3II revealed two distinct bands corresponding to autophagy-active LC3BII (lower) and autophagy-inactive LC3BI (upper). IB analyses of WDFY3 (~ 100 kD) and p62 (> 60 kD) were also performed in splenic Mφs of NTg and Tg^+/-ATG13^ mice. Beta-actin immunoblotting was performed as a loading control. **F** The relative densitometric analysis results were plotted after normalization to the respective β-actin bands. The white and gray bars represent the LC3B band densities of the NTg and Tg^+/-ATG13^ mice, respectively. (An unpaired t test was performed to test the significance of the difference in the means between groups; *p < 0.05 vs. the LC3BI of Tg^+/-ATG13^. NS = not significant. **G** DAB immunostaining of splenic sections of WDFY3 (rabbit; cat# Invitrogen, dilution 1:100) (*n* = 5/group) counterstained with hematoxylin. (inset) Enclosed zones are magnified (a = NTg, b = Tg). **H** WDFY3 + cells were counted in 5 different images from 5 mice/group following the quantification per sq.mm. of sections (unpaired t test; ***p < 0.005 = 0.002844221). **I **&**J** Representative flow cytometry analyses of WDFY3 (PE-tagged) and CD11b (FITC-tagged) cells (10000 gated events). **K** Histogram of the number of gated cells in the circled population. **L** The scatter bar graph shows the counts of CD11b + WDFY3 + cells in six analyses per group (unpaired t test shows ****p < 0.0005). The results are presented as the mean ± SD of three experiments

### Immunoblot analysis

Cells and tissue lysates were prepared with 5× Laemmli buffer, separated on 4–12% Tris–glycine gels, and transferred to nitrocellulose membranes (P/N 926–31,090; LI-COR Biosciences). The membranes were incubated overnight with primary antibodies, incubated for 2 h at room temperature with IRDye700/800-tagged secondary antibodies on an orbital shaker and imaged using an Odyssey Sa imager at 200 μm resolution.

**Fig. 3:**
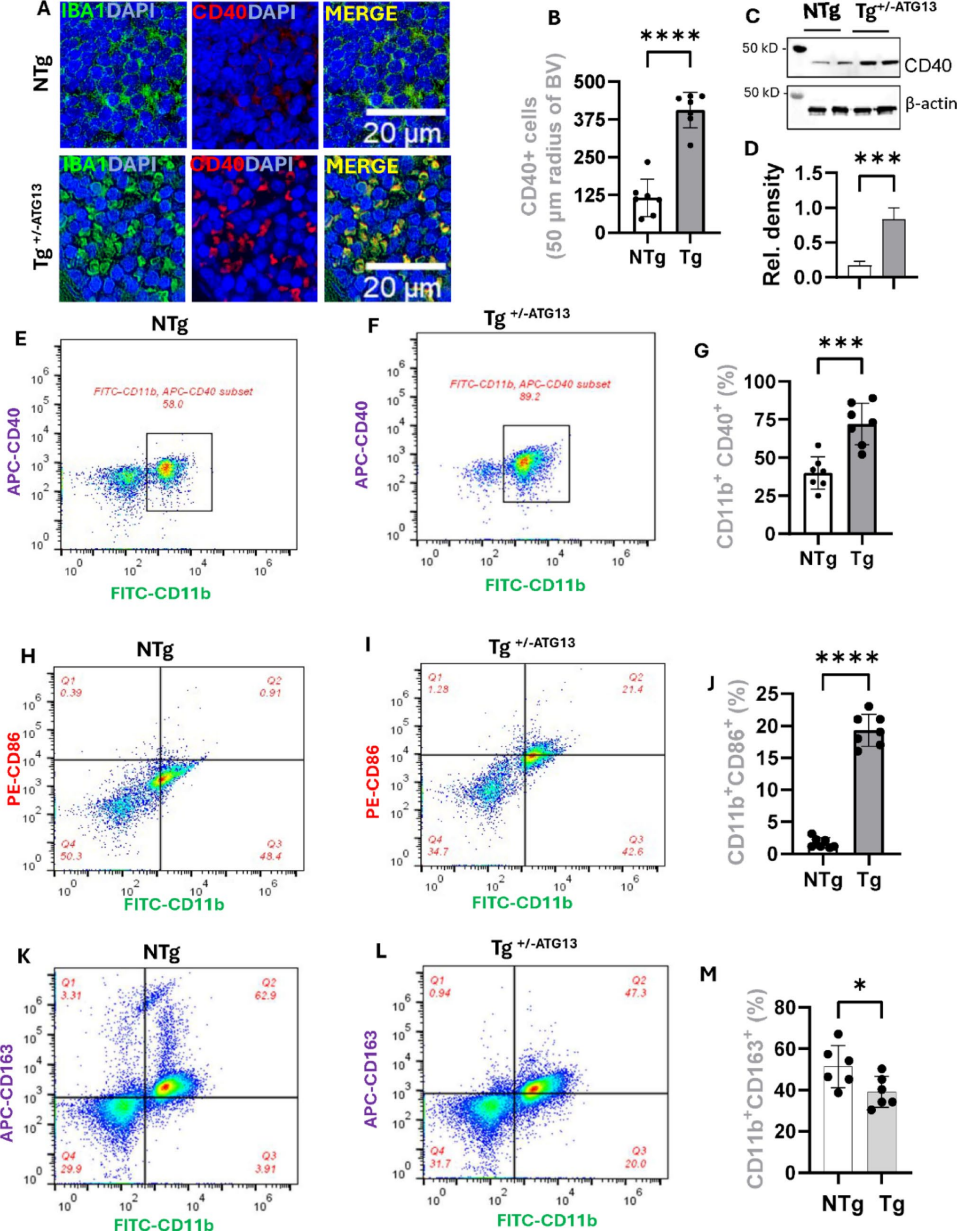
Polarization of M1Mφ cells in the spleens of Tg+/-ATG13 mice. **A** Dual IF analysis of CD40 (rabbit anti-CD40; Cat#; ProteinTech; dilution 1:100) and IBA1 (mouse anti-IBA1; Cat#; Invitrogen; dilution 1:100) in 5 μm thick paraffin-embedded spleen sections from 10- to 12-week-old male NTg and Tg+/-ATG13 mice (n=6/group). **B**Quantification of CD40-ir cells in the 50 μm radius of blood vessels in the red pulp zone was performed. An unpaired t test was performed to verify the significance of the difference in the means between groups, and the results are shown as ****p<0.0005. The results were confirmed after counting 7 independent images per group. **C**IB followed by **D** β-actin-normalized densitometric analyses; ***p<0.005 (unpaired t test) between groups. Dual flow cytometry of APC-labeled CD40 and FITC-labeled CD11b in purified Mφs isolated from 10- to 12-week-old **E**NTg and **F**Tg+/- ATG13 mice (n=6 male). **G**The quantification analysis of CD11b+ CD40+ population was performed followed by measuring the significance by unpaired t-test (***p<0.005) . The total number of gated events was 20,000/group. **H & I**Similarly, dual flow cytometry analysis of CD86 (PE-tagged; dilution 1:100) and CD11b (FITC-tagged; dilution 1:100) followed by **J** quantification (n=7 analysis/group) was performed (****p<0.0005; unpaired t test). **K & L** Dual flow cytometry analysis of CD163 (APC-tagged; dilution 1:100) and CD11b (FITC-tagged; dilution 1:100) followed by **M**quantification (n=7 analysis; ****p<0.0005 by unpaired t test) was performed on purified Mφ cells. The results are presented as the mean ± SD of three experiments

### Live cell phagocytosis assay

Approximately 80,000 adherent Mφ cells (counted in Countess™ automated counter) were added to 100 µL of serum-free DMEM supplemented with 20 µL of pHrodo™ green BioParticles™ Conjugates (Cat# P35365; ThermoFisher Scientific, Inc.) and then incubated at 37 °C for 15 min. Afterward, the conjugate media was discarded, and the reaction was stopped by adding chilled methanol followed by storage at -20 °C. Subsequently, the cells were imaged under the FITC filter (480 nm) of an Accuris fluorescence microscope. For flow cytometry, the cells were carefully scraped, centrifuged, and resuspended in 100 µL of FACS buffer supplemented with 20 µL of pHrodo™ green BioParticles™ Conjugates and other dye-tagged antibodies at the recommended dilution. After incubating at 37 °C for 15 min, the cells were washed and subjected to flow cytometry analysis.

**Fig. 4:**
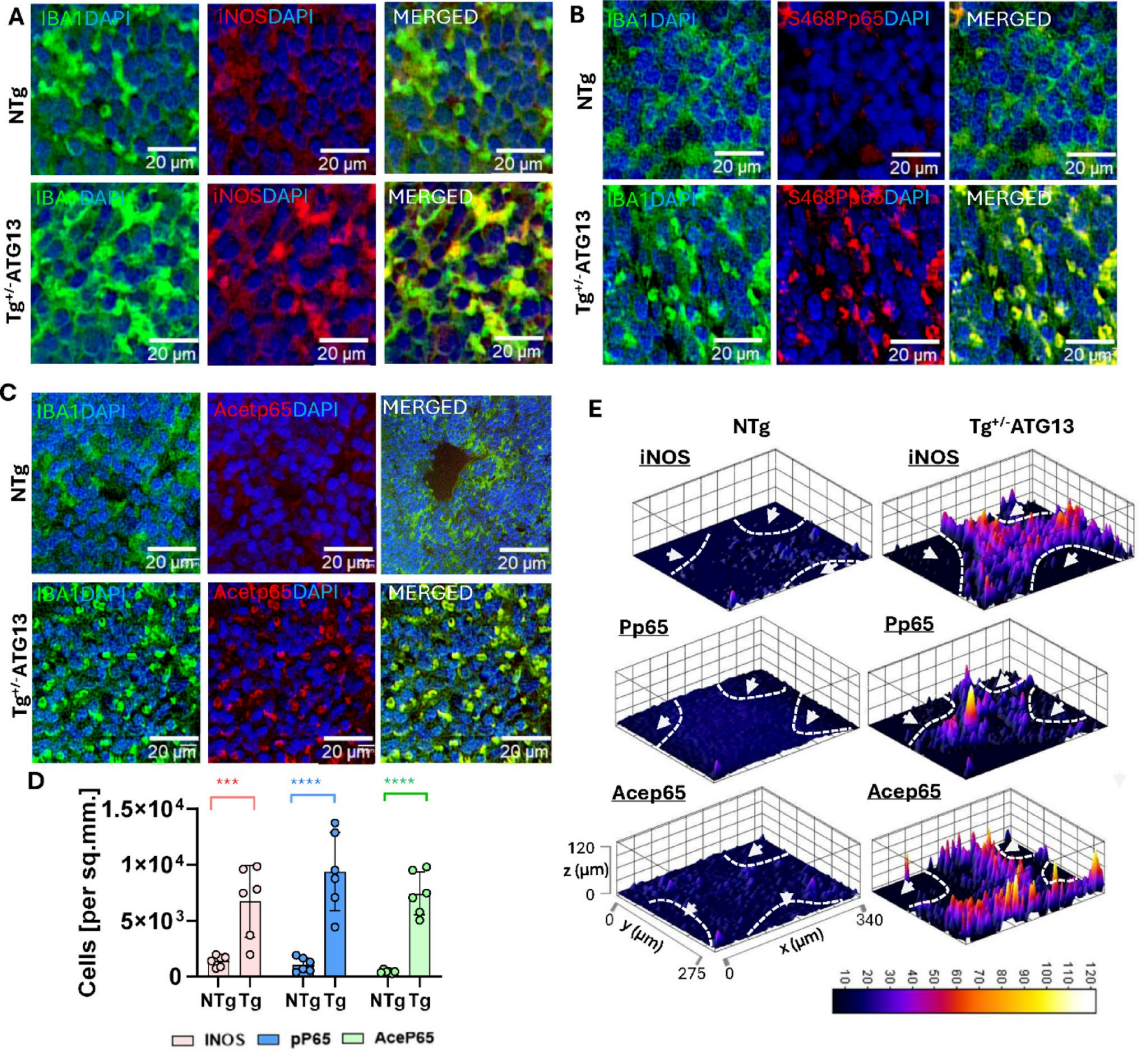
Functional characterization of M1Mφ cells in the spleens of Tg^+/−ATG13^ mice. Dual IF analysis of IBA1 (mouse anti-IBA1; Cat#; Invitrogen; dilution 1:100) with M1Mφ functional markers, including **A** iNOS (rabbit anti-CD40; Cat#; ProteinTech; dilution 1:100), **B** serine 468phospho (S468P) NFκB subunit p65, and **C** acetylated p65 in paraffin-embedded spleen sections of 10- to 12-week-old NTg and Tg^+/−ATG13^ mice (*n* = 6/group). **D** Quantification analyses of iNOS (pink bars), S468Pp65 (blue bars), and acetylated p65 (green bars) in *n* = 6 independent images. Unpaired t tests were used to test the significance of the mean results; ***p < 0.005 and ****p < 0.0005 versus NTg. **E** 3D surface plots (ImageJ software) were drawn to visualize the fluorescence intensities and the distribution of iNOS, S468Pp65, and acep65-ir cells in the red pulp regions. The red pulp regions were separated from the white pulp regions (arrowhead) by a dotted white line. A distinctively lower distribution of immunoreactive cells was observed in the white pulp regions. The results are presented as the mean ± SD of three experiments

### MitoSOX™ assay and kinetic study

Briefly, splenic Mφs (~ 100,000) cells were serum-starved for 6 h and concurrently treated with increasing doses (50 and 100 nM) of SBI-0206965 (Cat#SML1540; Sigma-Aldrich) or 100 nM BL-918 (Cat# AMBH93E4C342; Ambeed, Inc). After that, these cells were stained with MitoSOX™ green dye as recommended dilution ( Cat# M36005;ThermoFisher Sci), incubated for 30 min at 37^◦^C /5% CO_2_ incubator, and then imaged under FITC filter. In another case, the purified Mφs from NTg and Tg mice were subjected to a kinetic measurement at 488/510 nm wavelength at 1 min interval for 1 h.

### SiRNA transfection analysis

Mouse splenic Mφs were transfected with 25 pmol of sirt1 siRNA (Cat# 4390771; ThermoFisher Scientific) in serum-free medium using Lipofectamine 2000 according to the manufacturer’s protocol. After 4 h, serum was added, and at 24 h post-transfection, the cells were treated with 0.5 mg/mL LPS. Sirt1 expression and activity were assayed 2 h later.

### Sirtuin assay

For the SIRT1 activity assay, SIRT1 activity was measured in spleen lysates from transgenic and nontransgenic mice using the SIRT1 Activity Assay Kit (Fluorometric) (BPS Bioscience, Cat# 50081) following the manufacturer’s protocol. Briefly, spleens were harvested, rinsed in cold PBS, and homogenized in ice-cold lysis buffer (20 mM Tris-HCl (pH 7.5), 150 mM NaCl, 1 mM Na2EDTA, 1 mM EGTA, 1% Triton X-100, 2.5 mM sodium pyrophosphate, 1 mM β-glycerophosphate, 1 mM Na3VO4, and 1 µg/ml leupeptin with protease inhibitors) (Cell Signaling Technology Cat# 9803). The homogenates were centrifuged at 12,000 × g for 10 min at 4 °C, after which the supernatants were collected. The protein concentration was quantified using Bradford reagent (ThermoFisher Scientific, Cat# 1856209).

**Fig. 5:**
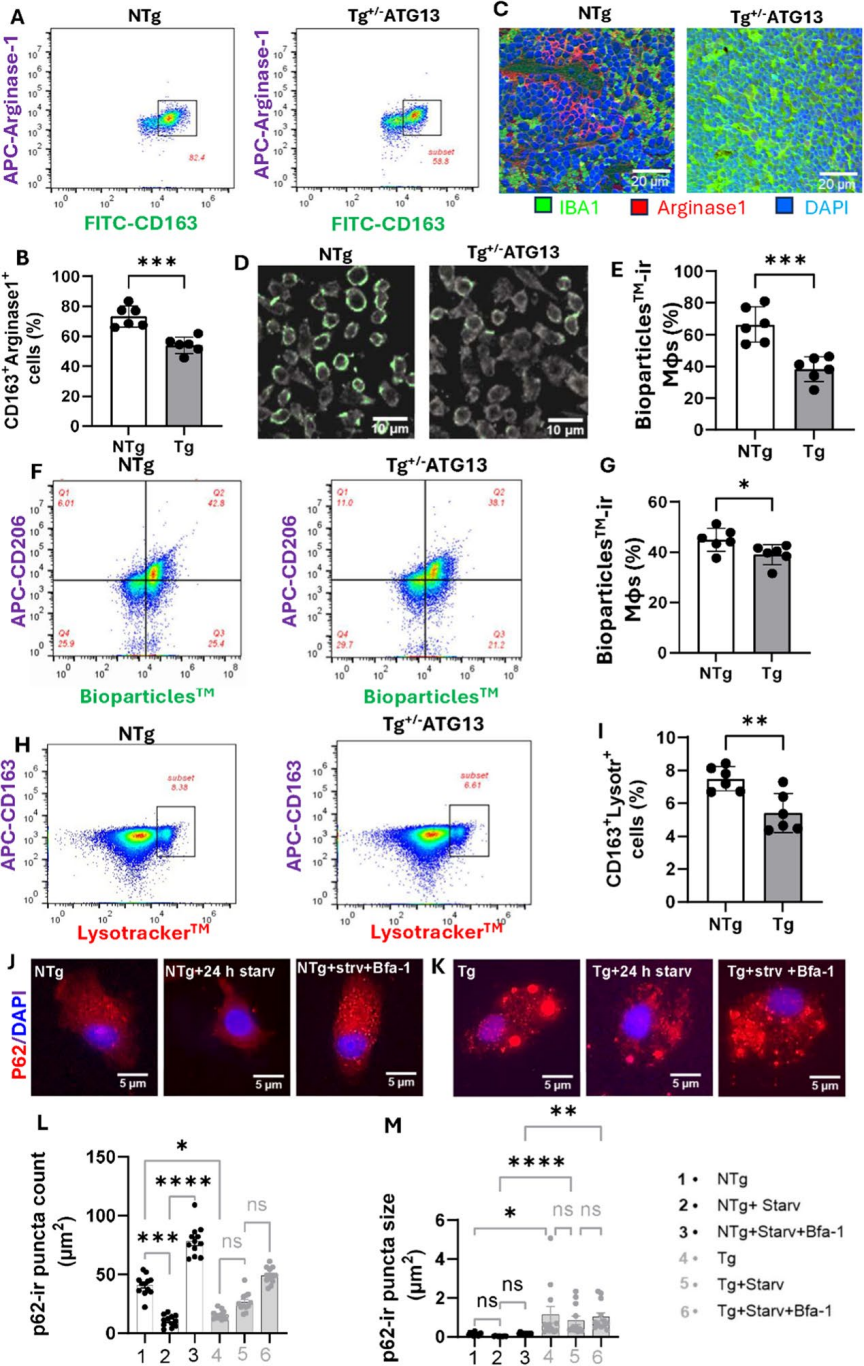
Functional characterization of the M2Mφ phenotype in splenic Mφ cells from Tg^+/−ATG13^ mice. **A** Dual IF analysis of the M2Mφ functional marker arginase-1 (APC-tagged) and the M2Mφ surface marker CD163 (FITC-tagged). The enclosed subsets show CD163 + arginase 1^+^ cells among the purified splenic Mφs. Total gated events = 10,000. **B** Scatter histogram displaying the number of CD163 + Arginase 1^+^ cells in *n* = 6 analyses/group. An unpaired t test was used to determine the significance of the difference in the means between groups, ***p < 0.005. **C** Paraffin-embedded splenic tissue sections of NTg and Tg^+/-^ATG13 were immunostained with IBA1 (green) and Arginase-1(red). Nuclei were stained with DAPI. **D** Phagocytosis assay of live Mφ cells by immunocytochemistry with pHrodo™ green conjugated BioParticles™ (Cat#P35381; ThermoFisher; 10^6^ cells in 10,000 Mφ cells). **E** Quantification of phagocytic cells in *n* = 6 images/group followed by an unpaired t test to verify the significance of the difference; ***p < 0.005. **F** Dual flow cytometry of CD206 (APC-tagged) and Bioparticles™ (pHrodogreen-tagged) followed by **G** quantification of Bioparticles™ (%)-conjugated ^CD206+^ cells (*n* = 6 analyses). An unpaired t test was used to verify the significance of the mean, and the results are indicated by *p < 0.05. Total gated events = 20,000. **H **Dual flow cytometry of CD163 (APC-tagged) and LysoTracker™ (red-DND99-tagged detected in PE filter; ThermoFisher; Cat# pHrodogreen-tagged) followed by **I** quantification of LysoTracker™ (%)-conjugated CD163^+ ve^ cells (*n* = 6 analyses). An unpaired t test was used to verify the significance of the mean, and the results are indicated by *p < 0.05. Total gate = 50,000. P62 IF analysis in **J** NTg and **K** Tg Mφs treated with 100 nM Bafilomycin A1 for 2 h under control and serum-starved condition (24 h). **L** Numbers and **M** size for p62-ir puncta were measured in 12 independent cells per group by ImageJ software, displayed in histogram analyses. The significance of mean was calculated by Kruskal-Wallis test, resulting in *p < 0.05, **p < 0.01, ***p < 0.005, ****p < 0.0001. The results are presented as the mean ± SD of three experiments

For the assay, 5 µL of 100 µM substrate, 5 µL of BSA (1 mg/ml), 5 µL of 50 mM NAD+, and 14.5 µl of SIRT assay buffer were added to each designated well of a 96-well black microplate. Then, 20 µL of spleen lysate (containing 20 µg of total protein) from the transgenic or nontransgenic mice was added. Each sample was run in triplicate. Then, 50 µL of SIRT assay developer (2x) was added, and the plate was incubated at room temperature for 15 min. Fluorescence was measured at Ex/Em = 385/485 nm at 60-second intervals for 30 min using a Victor™ X3 (PerkinElmer) microplate reader.

### SIRT2 activity assay

SIRT2 activity was measured in spleen lysates from transgenic and nontransgenic mice using the SIRT2 Activity Assay Kit (Fluorometric) (Abcam, Cat# ab156066) following the manufacturer’s protocol. Briefly, spleens were harvested, rinsed in cold PBS, and homogenized in ice-cold lysis buffer as described for the SIRT1 assay procedure. The tissue lysates were centrifuged at 12,000 × g for 10 min at 4 °C, after which the supernatants were collected. The protein concentration was quantified using Bradford reagent (ThermoFisher Scientific, Cat# 1856209).

For the assay, 25 µL of double distilled water, 5 µL of SIRT2 assay buffer, 5 µL of fluoro-substrate peptide, 5 µL of NAD + solution and 5 µL of developer solution were added to each of the designated wells of a 96-well black microplate. Then, 5 µL of spleen lysate (containing 20 µg of total protein) from the transgenic or nontransgenic mice was added. Each sample was run in triplicate. Fluorescence was measured at Ex/Em = 485/535 nm at 120-second intervals for 30 min using a Victor™ X3 (PerkinElmer) microplate reader.

### Seahorse mitochondrial OCR and glycolytic ECAR analyses

Measurements of mitochondrial oxygen consumption and glycolysis in Mφ cells were performed by using a Seahorse XF96 extracellular flux analyzer. Briefly, Mφs were isolated from the spleens of 10- to 12-week-old male NTg and age-/gender-matched Tg^+/−ATG13^ mice (*n* = 3/group), suspended in Agilent-recommended seahorse RPMI media (part # 103576-100), and seeded in a Seahorse XF96 96-well microplate (part #102959 − 10096) at a concentration of 200,000 cells/well. To ensure proper attachment at the bottom, the cells were spun down, and the media was replaced with fresh RPMI media. Oxygen was measured, and at the specified time points, solutions of different mitochondrial and glycolytic inhibitors were added. The data were displayed with Seahorse Wave software and then exported to GraphPad Prism using a dropdown menu under the export tab.

For the post-treadmill seahorse OCR and ECAR studies, age-matched (*n* = 3) males were subjected to treadmill exercise at 14 rpm for 15 min. After 4 h, the splenic Mϕs were isolated, counted, plated in OCR/ECAR media and immediately measured for the OCR/ECAR assay. The results were compared with those of splenic Mϕs from age-, sex- and genotype-matched mice (*n* = 3) not subjected to treadmill exercise.

**Fig. 6:**
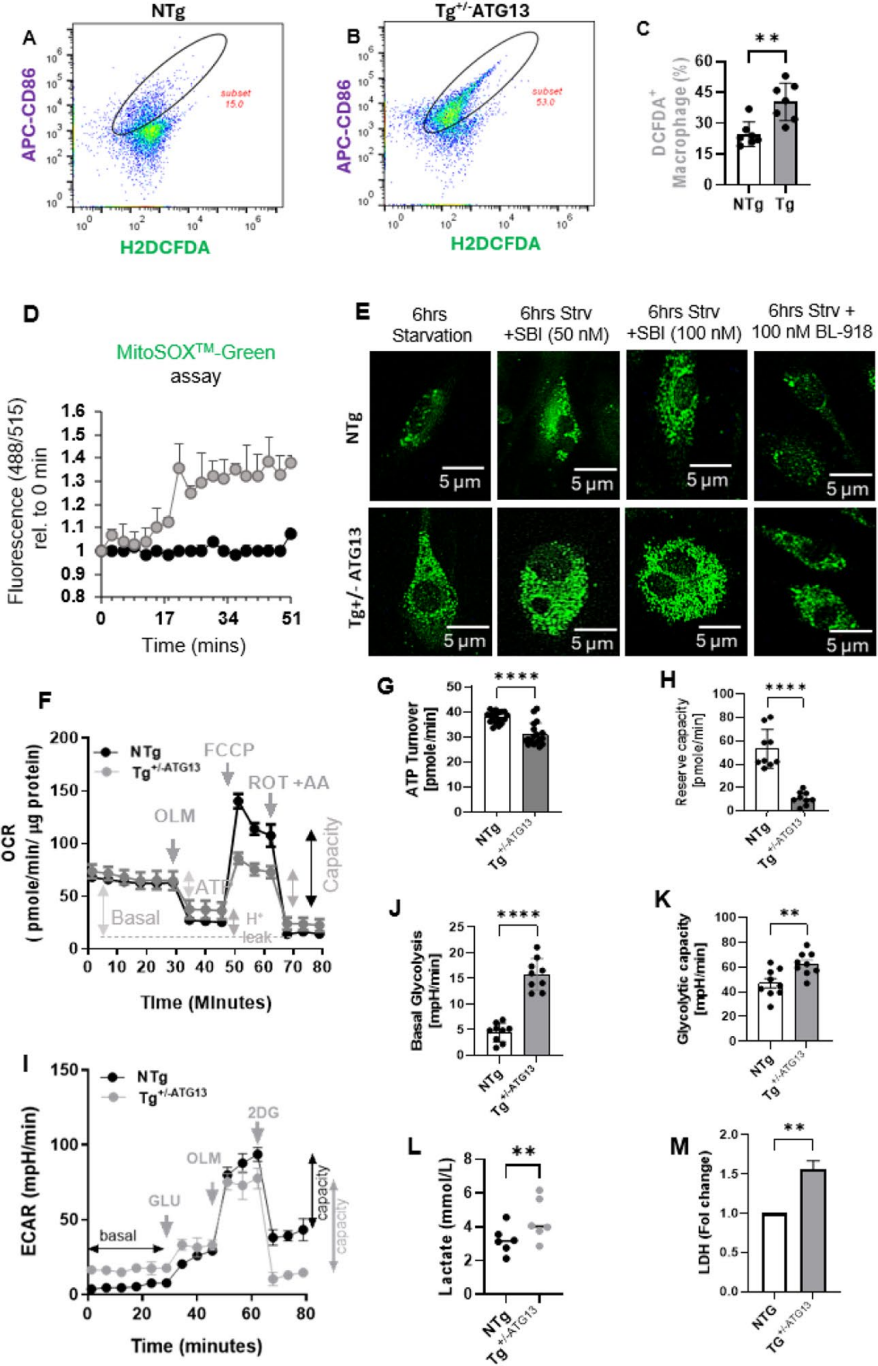
Production of ROS and evaluation of metabolic events responsible for ROS production in Mφ cells from Tg^+/−ATG13^ mice. FC analysis with the ROS sensor H2DCFDA (FITC-filter) in **A** NTg and **B** Tg Mφ cells. The enclosed ellipsoids are subsets of the ROS-producing population in purified splenic Mφs. Total gated events = 10,000. **C** Scatter histogram displaying the number of DCFDA + cells in *n* =7  analyses/group. An unpaired t test was used to determine the significance of the difference in the means between groups, with **p < 0.01. **D** The MitoSOX™ green kinetic assay was done to measure superoxide radicals in NTg and Tg^+/−ATG13^ Mφs (Gray = Tg; Black = NTg). **E **Fluorescence microscopy was done to visualize superoxide production after 6 h of starvation alone or with ULK-1 inhibitor SBI-0206965 (50 & 100 nM) or ULK-1 agonist BL-918 (100 nM). **F** The Seahorse metabolic assay of mitochondrial oxidative phosphorylation (OXPHOS) was evaluated with XF96 equipment (Agilent) by measuring the oxygen consumption rate (OCR) followed by visualization and analysis with Agilent Wave™ software. Approximately 200,000 cells were treated with OLM = Oligomycin (2 µg/mL); FCCP = Fluoro-carbonyl cyanide phenylhydrazone (2 µM), ROT = rotenone, and AA = antimycin A. **G** ATP utilization and **H** reserve capacity indicative of the capacity of performing OXPHOS under stress was evaluated by scatter histogram analyses. ****p < 0.0005 versus NTg based on an unpaired t test. **I** Glycolysis was evaluated by measuring the extracellular acidification rate (ECAR) in a Seahorse XF96 system and subsequent analysis via Wave_TM_ software. GLU = glucose, 2DG = 2 Deoxy-glucose. Scatter histogram analyses of **J** basal glycolysis and **K** glycolytic capacity. An unpaired t test revealed ****p < 0.0005 versus NTg. **L** NTg and Tg^+/−ATG13^ Mφs were serum-starved for 24 h followed by measuring lactate production in supernatants. An unpaired t-test displayed **p < 0.005 vs. NTg. **M** LDH release assay was measured by CyQUANT™ LDH assay (ThermoFisher) kit (paired t test **p < 0.01). The results are presented as the mean ± SD of three experiments

### Treadmilling, open-field, grip strength, and EMG analyses

Treadmilling, open-field gross movement and EMG recording were performed as previously described [19].

A Columbus XR4 mouse treadmill (S/n APM933AT-I) was used to assess muscle fatigue. The mouse was subjected on the motorized belt at 5 rpm for 1 min and then at 15 rpm for 14 min. The track’s end was blocked to prevent escape.

Following treadmill exercise, each mouse underwent gross movement assessment in an open-field acrylic arena (Stoelting Co; Cat # 60,100). The arena measures 40 cm on each side and features transparent walls as well as a detachable fiber base for cleaning. Movement was recorded using a Stoelting digital USB camera (Cat#10–000–332) mounted on the ceiling via a suspension bar. The camera was connected to ANY-maze video tracking software with a cable. For each trial, horizontal activity, total distance moved, movement time, resting time, and tracking plot were measured. Each recording began after a 2-minute acclimatization period.

For grip strength measurement, male Tg^+/−ATG13^ and NTg mice aged 10–12 weeks (*n* = 6) were tested using a rectangular grid attached to a console (Maze Engineers) that records grip force (Newton) over time, displayed via Grip TestUI (version 1.0.0.0) software. For baseline recording, mice were acclimatized on the grid for one minute, followed by grip strength assessment through a force versus time scatter plot over five minutes. Subsequently, the mice underwent treadmill exercise for 15 min at 14 rpm, rested for one day, and were then retested for grip strength. Results are presented as mean ± SEM.

EMG recordings were obtained from both nontransgenic (NTg) and Tg^+/−ATG13^ mice using AD Instruments PowerLab, amplified with Bio Amp PowerLab, and sampled at 2 kHz. The results were displayed in Chart software at 10–50 µV resolution, with reference values ranging from − 5 to 5 mV.

### Statistical analysis 

 The sample size *n* = 8 was calculated with the help of a statistical calculator based on a confidence interval of 0.95, margin of error of 5%, and population proportion = 0.5. The significance of the difference in means between groups was analyzed with GraphPad Prism 10 software by using an unpaired t test (parametric; 2 groups), a Mann‒Whitney U test (nonparametric; 2 groups), one-way ANOVA (parametric and one effector; more than 2 groups) or two-way ANOVA (parametric and two effectors; more than 2 groups) combined with Tukey’s HSD multiple comparison tool. The decision to perform either parametric or nonparametric tests was made after assessing the normality of the distribution of the Q‒Q plot, followed by the D’Agostino & Pearson test (*p < 0.05). The data are presented as the mean ± SEM, and a p value < 0.05 was considered to indicate statistical significance.

**Fig. 7:**
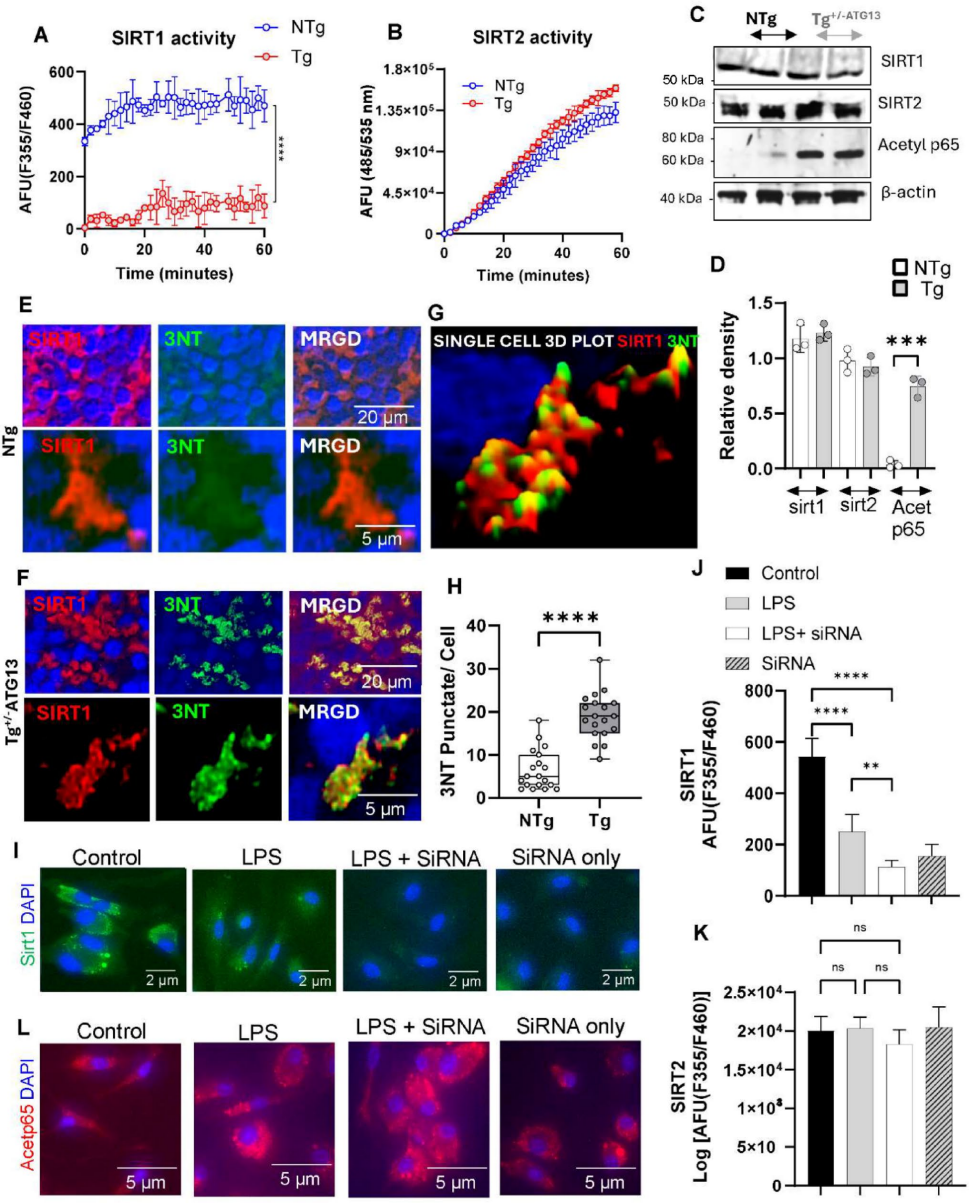
The ROS-mediated inhibition of Sirtuin-1, but not Sirtuin-2, may be responsible for inflammation. **A** A fluorescence-based (excitation: emission = 355/460 nm) enzyme assay of sirtuin-1 (SIRT1) in splenic Mφs. The assay was performed as instructed by the manufacturer (BPS Biosciences) using a cell lysate containing 1 µg of total protein. **B** Fluorescence-based sirtuin-2 assay performed according to the manufacturer’s protocol (Abcam) with 1 µg of protein derived from the cell lysate of Mφ cells. **C** IB analyses followed by **D** densitometric analyses of Sirt-1/2, acetylated p65, and β-actin in splenic Mφ cells from NTg and Tg^+/-ATG13^ mice (*n* = 3 per group). Dual IF analyses of SIRT-1 (red) and 3-nitrotyrosine (3NT; green) in the spleens of 10- to 12-week-old **E** NTg and **F** Tg^+/-ATG13^ mice (*n* = 6/group). **G** 3D visualization of a single cell (ImageJ) showing the interaction between SIRT1 and 3NT. **H** Quantification followed by scatter boxplot analysis of the total number of 3NT-ir puncta per cell in 19 cells/group. The unpaired t test indicates ****p < 0.0005 versus the control. **I**Tg splenic macrophages were transfected with siRNA against *sirt1 *(250 pmol; Cat # AM16708; Thermo Fisher Scientific, MA), treated with 0.5 mg/mL LPS for 2 h, and then immunostained with a Sirt1 antibody to determine the efficiency of the siRNA. Enzyme activity assay of **J** SIRT1 and **K** SIRT2 after 30 min of incubation with siRNA-transfected and non-transfected cell lysates (1 µg protein). **L** IF analysis of acetylated p65 in Tg splenic Mφ cells transfected with Sirt1 siRNA and then treated with 0.5 mg/mL LPS. The results are presented as the means ± SEMs of three different experiments

## Results

### Depletion of the ATG13 gene in Tg-ATG13 mice inhibited macroautophagy in splenic Mφs

 Previously, our research [19] demonstrated that ATG13 repressor mice (Tg-ATG13) with lacZ repressor-mediated suppression of *atg13*, an essential autophagy gene, strongly impaired autophagy and induced inflammatory demyelination in muscle-serving nerve fibers. However, the underlying molecular mechanism was unclear. To determine the molecular role of the atg13 gene, we generated cre-loxP conditional knockout (KO) mice (Tg^+/−ATG13^) (Fig. 1A) with a hemizygous deletion of the critical exon 5 of the *atg13* gene (Fig. 1B). These male Tg^+/−ATG13^ mice had significantly enlarged spleens, indicating that *atg13* gene-depleted splenic cells may be associated with altered immune metabolism. To understand the role of ATG13 in metabolic deficits in immune cells, especially in macrophages (Mφ), we first performed dual immunostaining for ATG13 and the inflammatory Mφ marker IBA1 (Fig. 1C) in the spleen. Dual IF analysis together with Pearson correlation analysis (Fig. 1D) of splenic sections followed by IB analysis of purified splenic Mφ cells revealed that hemizygous depletion of the atg13 gene in Tg^+/−ATG13^ significantly reduced ATG13 expression (Fig. 1E and F) in IBA1-ir inflammatory Mφ cells but not in nontransgenic (NTg) Mφ cells, indicating that depletion of the *atg13* gene may promote inflammation in Mφ cells. Interestingly, the expression of another autophagy protein, ATG101(Fig. 1E and G), which forms a complex with ATG13 to initiate autophagosome formation, did not change significantly.

To understand the molecular role of atg13 in regulating the cellular metabolism of autophagy in Mφ cells, the following assays were performed. *First*, a dual IF analysis (Fig. 2A) of the pan-Mφ marker CD11b and the general autophagy marker LC3 indicated that the LC3 level was significantly lower in Mφ cells of the Tg^+/−ATG13^spleen compared to same of NTg mice. *Second*, dual flow cytometry analyses (Fig. 2B-D) of CD11b and LC3 also revealed a strong and significant reduction in the LC3-ir cells in the splenic Mφ cells of Tg^+/−ATG13^ mice. *Third*, the functional significance of LC3 upregulation in autophagy was further evaluated by IB analysis (Fig. 2E) followed by quantification of the level of LC3II compared with that of LC3I (Fig. 2F) in splenic Mφ cells, which indicated a significant reduction in LC3II in Tg^+/−ATG13^Mφ cells. *Fourth*, to evaluate the role of ATG13 in overall autophagic flux, we performed p62 IB analysis in splenic Mφs of both NTg and Tg mice. As reported elsewhere [15], The IB analysis revealed that the level of p62 is significantly high in Tg, compared to NTg Mφs, suggesting that there was a strong impairment of autophagy in Tg^+/− ATg13^ splenic Mφs. *Fifth*, IB analysis (Fig. 2E; *middle panel*) and DAB immunostaining (Fig. 2G) of the macroautophagy marker WDFY3 were performed, followed by quantification analysis (Fig. 2H), which revealed a significant loss of autophagy in Tg^+/−ATG13^Mφ cells. *Sixth*, a flow cytometry analysis (Fig. 2I and J) coupled with a quantification analysis (Fig. 2K and L) further indicated that the WDFY3-ir population was significantly reduced in the purified Tg^+/−ATG13^Mφ cells. *Finally*, the reduction in WDFY3 in purified splenic Mφ cells was confirmed by IB analysis, which revealed that genetic suppression of the *atg13* gene indeed inhibited autophagy in Mφ cells.

**Fig. 8:**
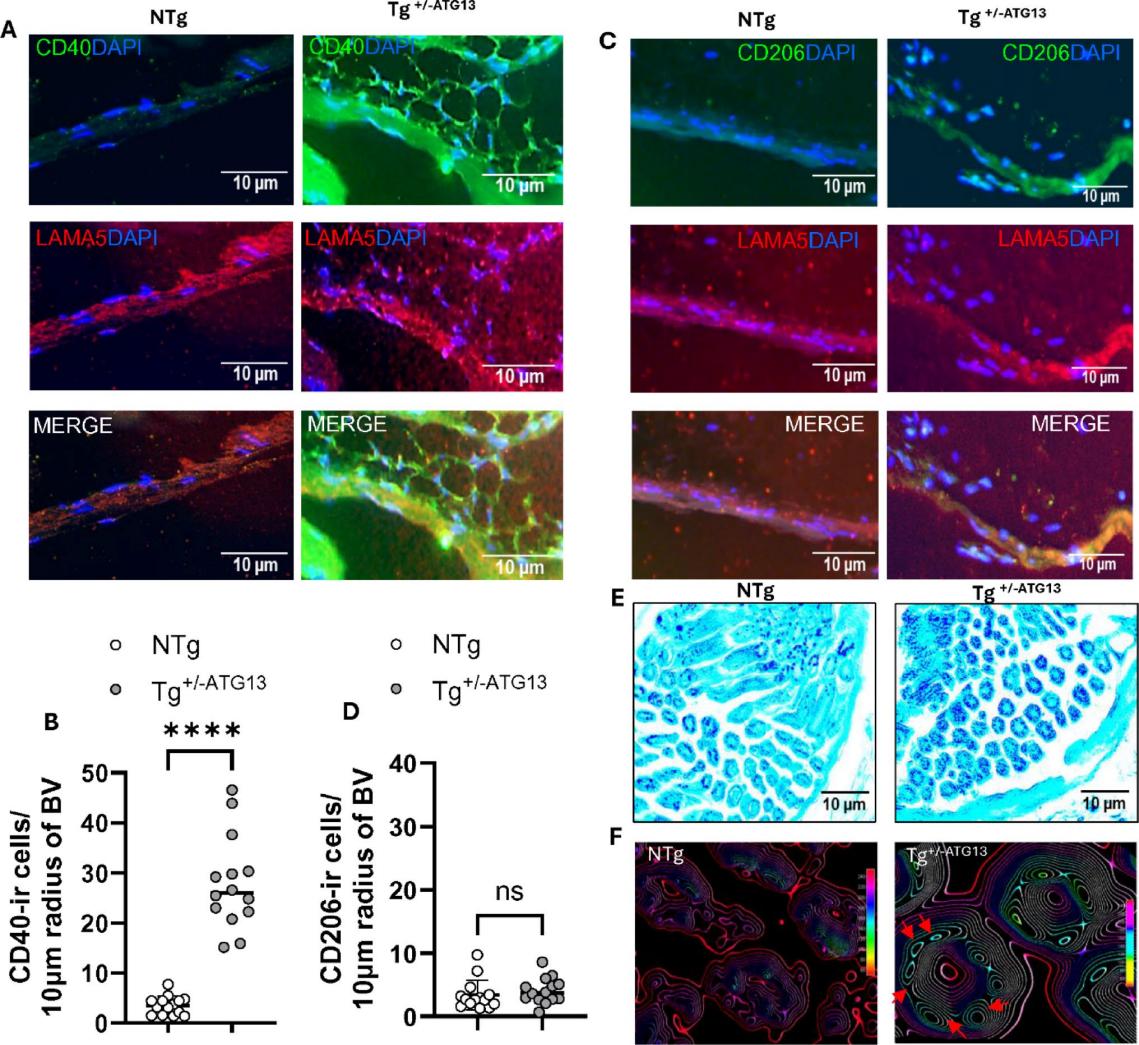
ATG13 ablation mediates perivascular infiltration of M1Mφ cells and may compromise myelin integrity. **A** Dual IF analysis of the M1Mφ marker CD40 (green; dil 1:100) and the blood vessel endothelium marker laminin alpha-5 (LAMA5; red; 1:100). **B** Scatter histogram analyses of CD40-ir cells at a 10 μm radius from the outer wall of the blood vessel. The unpaired t test represents the significance of the difference in the mean between two groups, with ^****^p < 0.0005: *n* = 13 vessels/group. **C** Dual IF analysis of the M2Mφ markers CD206 (green) and LAMA5 (red). **D** Scatter histogram analyses of CD206-ir cells at a 10 μm radius from the outer wall of the blood vessel. NS = not significant according to the count (*n* = 13 vessels/group). **E** LFB staining of myelin (lighter blue) in muscle-serving nerve bundles counterstained with cresyl violet (deep blue) in the biceps femoris muscle of NTg and Tg ^+/−ATG13^ mice (*n* = 5/group). **F** 3D surface plot (ImageJ) showing axonal fibers (cyan circles labeled with red arrowheads) with decreased myelin integrity. The results are presented as the mean ± SEM of three different experiments

### The effect of ATG13 gene suppression on the polarization of M1Mφ cells

 How does the suppression of the atg13 gene affect Mφ cell properties? During inflammation, Mφs acquire inflammatory M1 properties, which can be evaluated by analyzing the expression of a wide range of surface proteins. One such traditional M1 surface protein, CD40, was immunolabeled (Fig. 3A), as was the inflammatory marker IBA1 in the splenic tissue of Tg^+/−ATG13^ and NTg mice. Interestingly, both IBA1 and CD40-ir cells (Fig. 3B) were significantly upregulated in the spleens of Tg^+/−ATG13^ mice. These results were further confirmed by IB analysis (Fig. 3C) of purified Mφs followed by relative densitometric analysis (Fig. 3D) and flow cytometry (Fig. 3E-F) followed by quantification (Fig. 3G). The role of *atg13* depletion in the acquisition of the M1 phenotype was further confirmed by dual flow cytometry analysis of another M1 marker, CD86, with CD11b (Fig. 3H-I), followed by quantification analysis (Fig. 3J). The polarization of Mφ to the M1 phenotype has often been shown to be associated with deviation from the anti-inflammatory M2 phenotype. To test this possibility, we performed dual flow cytometry analysis of the M2 marker CD163 and the pan-Mφ marker CD11b (Fig. 3K-L**)** in purified splenic Mφs followed by quantification analysis (Fig. 3M). These results clearly indicate that there is a significant reduction in the M2 surface properties of the Mφ cells from the Tg^+/−ATG13^ spleen. Collectively, our results indicate that suppression of the *atg13* gene strongly induces polarization of Mφ cells toward the M1 phenotype.

Next, we investigated whether atg13 depletion stimulated the function of M1Mφs. Functionally, M1Mφ cells are associated with the upregulation of inflammatory mediators such as inducible nitric oxide synthase (iNOS) [28] as well as the phosphorylation of serine 468 (S468P) and the acetylation of the p65 subunit of nuclear factor kappa B (NF-κB) [29–31]. A series of dual IF studies indicated that IBA1-ir cells from Tg^+/−ATG13^ mice strongly expressed iNOS (Fig. 4A), S468P p65 (Fig. 4B), and acetylated p65 (Fig. 4C). These results were further corroborated by quantification analysis (Fig. 4D). To map the distributions of these inflammatory cells and the expression patterns of these mediators, we next performed surface plot analyses (Fig. 4E). Accordingly, we observed that the background-adjusted mean fluorescence intensities of iNOS, S468Pp65, and acetylated p65 were significantly greater in the Tg^+/−ATG13^ spleen than in the NTg spleen. Additionally, these Mφ cells were found to be specifically distributed in the red pulp but not in the white pulp (as indicated by arrowheads within the dotted line suggesting the marginal zone) region of the spleen. This observation suggested that depletion of the *atg13* gene led to the polarization and localization of M1Mφ cells near the vasculature of splenic tissue, potentially contributing to a systemic inflammatory response primarily due to the activation of NF-κB.

Does the suppression of *atg13* inhibit the function of M2Mφ cells? Given that our results indicate that *atg13* suppression may impede the polarization of M2Mφ cells, we proceeded to investigate whether the associated metabolic functions of M2Mφ cells are similarly inhibited. *First*, the neutralization of nitric oxide by arginase, a key metabolic event in M2Mφ cells, was evaluated via flow cytometry. Accordingly, dual flow cytometry (Fig. 5A) of arginase with the M2Mφ marker CD163 indicated strong suppression of CD163^+^Arginase^+^ cells (Fig. 5B) in purified splenic Mφ cells from Tg^+/−ATG13^ mice compared to those from NTg mice. Moreover, dual IF analyses of arginase and IBA1 (Fig. 5C) demonstrated that there was a strong reduction in arginase in IBA1-ir Mφ cells from the Tg^+/−ATG13^ spleen. Another functional property of M2Mφs is phagocytosis, which can be evaluated by a zymosan green assay in which live Mφs are fed green fluorescence-tagged zymosan particles. A fluorescence-based zymosan engulfment assay (Fig. 5D) followed by counting (Fig. 5E) and then a dual flow cytometry assay of zymosan green along with the M2Mφ marker CD206 (Fig. 5F-G) clearly demonstrated that atg13 depletion strongly attenuated the phagocytic ability of isolated splenic Mφ cells from Tg^+/−ATG13^ mice. Lysosomal function, an indirect indicator of autophagy, was significantly suppressed in Tg^+/−ATG13^ splenic Mφs, as determined by dual flow cytometry analysis of LysoTracker and CD163 (Fig. 5H) followed by quantification analysis (Fig. 5I). Impaired autophagosome formation doesn’t directly indicate lysosomal dysfunction, but depletion of ATG13 may lead to the defective protein and organelle buildup that may hinder lysosomal function. To test this, NTg (Fig. 5J) and Tg Mφs (Fig. 5K) were serum-starved for 24 h to induce autophagy, then treated with 100 nM bafilomycin A1 (Bfa-1) for 2 h to block lysosomal fusion. p62 IF analysis showed higher basal p62 in Tg Mφs than NTg Mφs. Notably, p62 puncta were observed to be larger in Tg Mϕs compared to NTg, which suggests defective protein accumulation indicative of impaired basal autophagy. Serum starvation reduced p62, and Bfa-1 treatment strongly increased p62 in NTg Mφs, while Tg Mφs had a slight decrease with starvation and moderate increase with Bfa-1 suggesting that a moderate impairment lysosomal fusion is also evident in Tg Mφs. Quantitative analyses were performed to analyze numbers (Fig. 5L) and size (Fig. 5M) of p62-ir puncta. Our findings show that under basal conditions, NTg displayed significantly more p62-ir puncta, while Tg Mϕs had much larger ones. This suggests that genetic depletion of atg13 not only reduces autophagosome formation (resulting in fewer puncta) but may also lead to abnormal protein accumulation. Starvation induced autophagy in NTg, but not in Tg Mφs. Furthermore, Bfa-1 treatment similarly blocked lysosomal fusion of p62-ir vesicles in both NTg and Tg Mφs, indicating that lysosomal fusion of autophagic vesicles might not be directly controlled by atg13 depletion. Overall, these results suggest that although lysosomal function is somewhat compromised, genetic depletion primarily impacts autophagosome formation in Tg Mφs. These findings collectively indicate that suppression of the *atg13* gene leads to increased expression of M1 surface markers, affects metabolic functions associated with M2 macrophages, and induces the inflammatory characteristics of splenic macrophages.

**Fig. 9:**
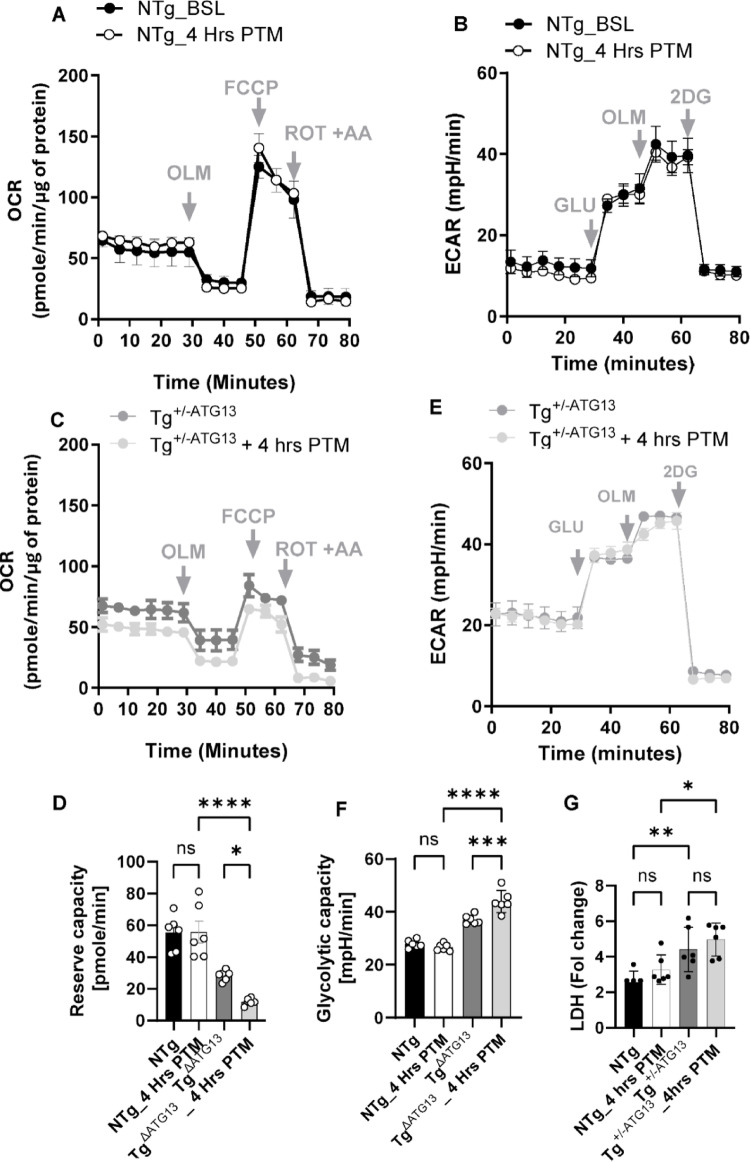
The post-treadmill exacerbation of mitochondrial energy metabolism in Tg^+/−ATG13^ mice. **A** Mitochondrial OCR of purified splenic Mφs from the NTg without treadmill exercise (BSL = baseline) and after 4 h post-Treadmill exercise (PTM) (*n* = 3/group). **B** Mitochondrial OCR of splenic Mφs from Tg^+/−ATG13^ mice before and 4 h after treadmill exercise (*n* = 3 per group). **C** Spare or reserve capacity, indicative of the ability of mitochondria to restore OXPHOS after stress, was measured in all four groups. Black bars = NTg without a treadmill, white bars = NTg after 4 h of treadmill exercise, dark gray bars = Tg without a treadmill, and lighter gray bars = Tg after 4 h of treadmill exercise. Two-way ANOVA (effectors: genotype and stressor) was adopted, followed by multiple comparison analyses to monitor the significance of the difference in the means between groups. ****p < 0.0005, *p < 0.05, and ns = not significant. Glycolysis was measured in enriched Mφs and subsequently compared between **D** NTg without a treadmill and 4 h PTM, as well as between **E** Tg^+/−ATG13^ without a treadmill and 4 h PTM ( *n* = 6 per group). **F** The glycolytic capacity, which is indicative of restorative ability after stress, was measured in all four groups. Two-way ANOVA was performed to test the significance of differences in means between groups. ****p < 0.0005, and ns = not significant. **G** LDH assay was performed in cell lysate as directed in CyQUANT™ LDH assay kit (ThermoFisher). Two-way ANOVAfollowed by multiple comparison analyses revealed *p < 0.05, **p < 0.01 and ns = not significant versus respective controls. The results are presented as the mean ± SEM of three different experiments

### Genetic ablation of ATG13 impairs redox metabolism in mitochondria resulting in the activation of NFκB via nitrotyrosination of the SIRT1 enzyme

 Next, we investigated the molecular mechanism through which the ablation of *atg13* gene induces M1Mφ polarization. The atg13 ablation-mediated loss of autophagy may directly compromise the mitochondrial function of energy metabolism, resulting in the production of ROS. In fact, the splenic M1Mφ cells of Tg^+/−ATG13^ mice exhibited increased ROS production, as indicated by dual flow cytometry analysis of the ROS sensor DCFDA and the M1 marker CD86 (Fig. 6A-C), suggesting a potential deficit in mitochondrial energy metabolism in Tg^+/−ATG13^ Mφ cells. While DCFDA assay measures the total ROS, mitochondrial ROS is primarily superoxide radicals [32], which was measured by MitoSOX™ green assay as described under method section. A real-time production of superoxide radicals (Fig. 6D) demonstrated that Tg^+/−ATG13^ splenic Mφs produced significant superoxide ROS compared to NTg controls. To investigate the direct impact of ATG13 depletion on mitochondrial superoxide production, macrophages were treated with the ULK1 (unc-51 like kinase 1) inhibitor SBI-0206965 (Sigma Aldrich), which effectively blocks residual ATG13 activation in hemizygous Tg^+/− ATG13^ splenic macrophages. Accordingly, our results indicated that SBI-mediated inhibition of ATG13 in Tg^+/−ATG13^ macrophages led to a more pronounced superoxide signal compared to NTg macrophages (Fig. 6E). Conversely, pharmacological activation of ULK-1 using BL-918 (100 nM), which stimulates ATG13 activity, normalized the superoxide radical formation in serum-starved NTg Mφs. Nevertheless, due to the hemizygous deletion of the atg13 gene in Tg^+/−ATG13^ Mφs, BL-918 was unable to fully normalize superoxide radical levels to those observed in NTg macrophages. These findings underscore the essential role of ATG13 in regulating mitochondrial ROS production. Our results indicate that increased ROS production in Tg mice may be due to the mitochondrial impairment of energy metabolism.

To explore the direct role of *atg13* gene ablation in mediating mitochondrial deficits in energy metabolism, we next performed a Seahorse oxygen consumption assay (Fig. 6F) with different mitochondrial inhibitors as described elsewhere [33]. Interestingly, the mitochondrial oxygen consumption rate (OCR) measurement assay in purified splenic Mφ cells displayed no difference in basal respiration (before oligomycin or OLM treatment). However, there was a significant decrease in ATP turnover (Fig. 6G) in Tg^+/−ATG13^Mφ cells, which was indicative of decreased bioavailability of ATP compared to that in the splenic Mφ cells of NTg mice. Additionally, Tg^+/−ATG13^Mφ cells exhibited a significant reduction in reserve capacity (Fig. 6H), indicating increased susceptibility to impaired mitochondrial energy production during stress. Next, we performed a glycolysis assay (Fig. 6I) in Mφs by measuring the extracellular acidification rate (ECAR) using different glycolytic inhibitors as described elsewhere [34]. Both basal glycolysis (Fig. 6J) and the glycolytic reserve (Fig. 6K) analyses indicated that genetic ablation of the atg13 gene in Tg^+/− ATG13^ mice increased glycolysis in Mφ cells under both basal and stress conditions. Glycolysis induction is necessary to maintain the inflammatory phenotypes of M1Mφ. While ECAR assesses the glycolytic activity of live cells, it serves as a proxy for lactate production because the acidification took place due to lactate release. Subsequently, lactate was measured (Cat# ab65331; Abcam) in Mφs after 24 h of serum starvation. Quantitative analysis **(**Fig. 6L) showed that Tg Mφs released significantly more lactate than NTg Mφs, indicating higher glycolytic efficiency in Tg Mφs, potentially as an adaptive response to mitochondrial impairment and support of inflammatory phenotypes. Furthermore, a notable and substantial elevation (Fig. 6M) in lactate dehydrogenase (LDH) activity was observed in Tg Mφs relative to NTg Mφs.

Collectively, our metabolic assays showed that *atg13* gene ablation impairs basal energy metabolism and ATP production. Glycolysis and lactate production increased in Tg^+/−ATG13^ Mφs, likely compensating for mitochondrial deficits and maintaining inflammatory function. Moreover, these impairments may worsen under stress.

**Fig. 10:**
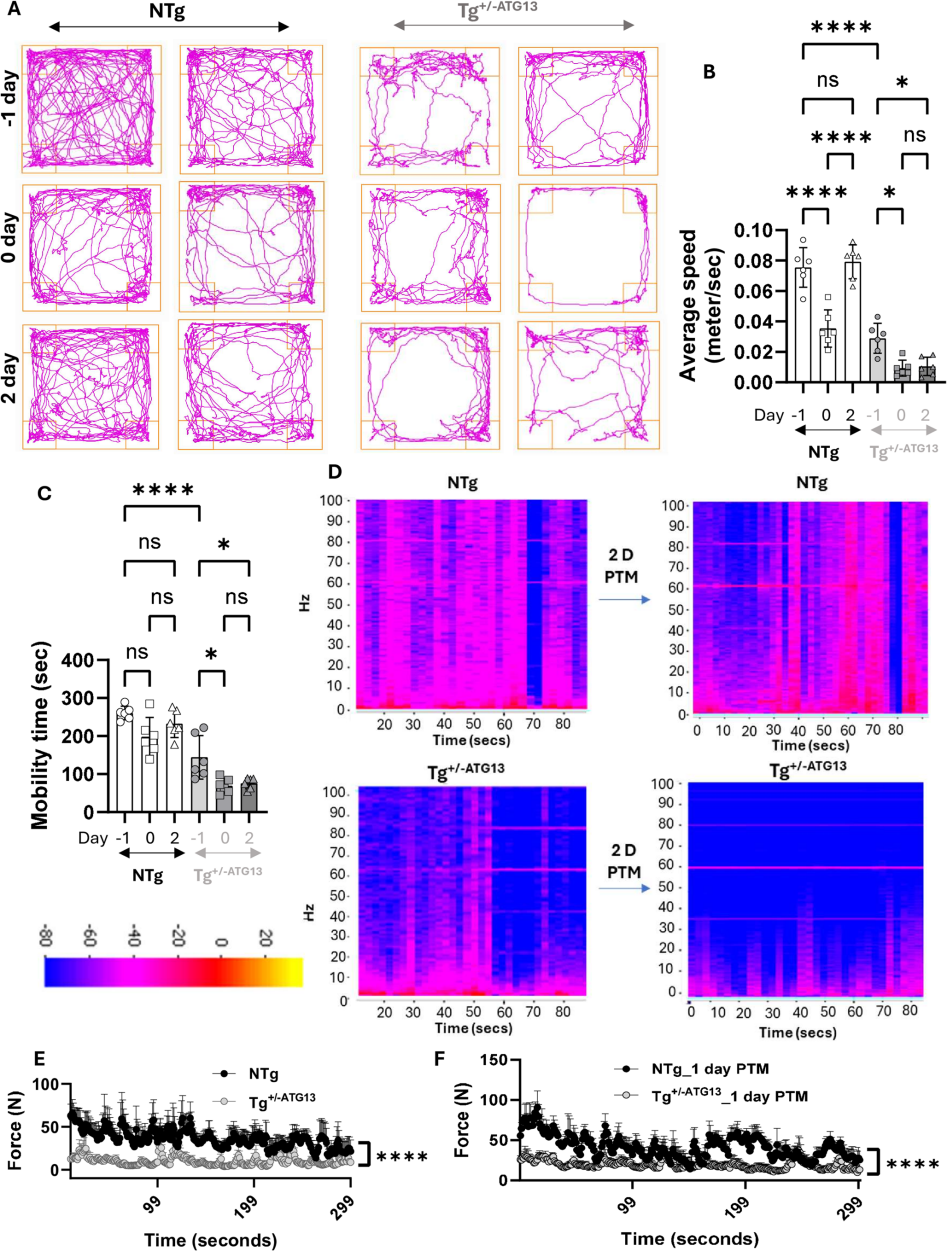
Muscle fatigue in Tg+/−ATG13 mice after treadmill exercise. **A** Representative trackplots indicating the gross movement of both NTg (left) and Tg+/−ATG13 (right) mice (10–12 weeks old) on the day before (-1 day), immediately after (0 day), and 2 days after treadmill exercise (4–14 rpm for 15 min). **B** Average speed and **C** mobility time were calculated at different time points during treadmill exercise. Two-way ANOVA followed by multiple comparison analysis (GraphPad Prism) was used to analyze the significance of differences in the means between groups. ****p < 0.0005 and *p < 0.05. **D** EMG recordings followed by the summation of frequencies were plotted as a heatmap. The all-limb grip strength measurements were performed **E** before and **F** one day after single-session treadmill (14 rpm /15 mins) exercise in a digital meter as described under method section. The recording was performed over 5 min in n = 6 NTg and Tg+/−ATG13 mice. The results are presented as the mean ± SEM of three different experiments

Next, we investigated how molecular deficits in energy metabolism contributed to the inflammatory properties of splenic Mφ cells in Tg^+/−ATG13^ mice. Our results (Fig. 4) indicated that acetylation of p65 was markedly increased due to the ablation of *atg13*. Recent studies have suggested that a class of cellular deacetylases known as sirtuins (SIRTs), especially isoform 1 (SIRT1) [35] and 2 (SIRT2) [36], regulate the deacetylation of p65 [37] and are therefore known for their roles in the suppression of inflammation. Interestingly, we observed that the activity of SIRT1 (Fig. 7A), but not SIRT2 (Fig. 7B), markedly decreased in Tg^+/−ATG13^ splenic Mφ cells. However, the cellular expression of both SIRT1 and SIRT2 was not altered by ATG13 depletion, as confirmed by IB (Fig. 7C) and subsequent relative densitometric analyses (Fig. 7D). The reduction in SIRT1 enzymatic activity may be attributed to post-translational modifications, such as nitrosylation, potentially arising from elevated oxidative stress within the mitochondria. To explore this possibility, we performed dual IF analyses of SIRT1 and 3-nitrotyrosine (Fig. 7E-F) followed by 3D colocalization mapping (Fig. 7G). Interestingly, the notable colocalization of SIRT1 and 3-nitrotyrosine in the Tg^+/−ATG13^ spleen (Fig. 7H) suggested that the loss of function of SIRT1 may be attributed to nitrosylation of tyrosine residues in response to oxidative stress following depletion of the atg13 gene. To further confirm these findings, we performed siRNA-mediated silencing of the *sirt1* gene in splenic Mφs via a commercially available (ThermoFisher) siRNA. Accordingly, SIRT1 expression (green puncta; Fig. 7I) and activity (Fig. 7J), but not SIRT2 expression (Fig. 7K), were markedly reduced in Sirt1 siRNA-transfected, LPS-activated Tg^+/−ATG13^ splenic Mφs, indicating effective Sirt1 gene silencing via the siRNA strategy. Interestingly, we observed that silencing of the sirt1 gene strongly upregulated acetylated NFκB in LPS-activated Tg^+/−ATG13^ Mφs (Fig. 7L), suggesting that the absence of ATG13 critically downregulated the enzymatic activity of SIRT1 to stimulate the activation of NFκB.

Previously, our study [19] revealed that suppression of the atg13 gene induces muscle fatigue in mice. To explore the underlying molecular mechanism involved, we investigated whether there was an increase in the infiltration of these polarized M1Mφ cells into the muscle vasculature of Tg^+/−ATG13^ mice. Interestingly, dual IF analysis of the blood vessel endothelium marker laminin alpha5 (LAMA5) and the M1Mφ marker CD40 (Fig. 8A) following a quantitative measurement at the 10 μm periphery of the blood vessel tract (Fig. 8B) revealed that there was upregulated infiltration of CD40-ir Mφ cells. However, when immunostained (Fig. 8C) and quantified (Fig. 8D) with the M2Mφ marker CD206, these cells displayed no immunoreactivity, suggesting that the infiltrated Mφs were primarily of the M1 rather than the M2 phenotype. Furthermore, LFB staining (Fig. 8E), which was used to evaluate myelin integrity in the nerve bundle of skeletal muscle (biceps femoris), demonstrated that genetic depletion of the atg13 gene significantly reduced myelin thickness (cyan) and increased the number of exposed axonal fibers (deep blue). A 3D surface plot (Fig. 8F) was drawn using ImageJ software to quantify the mean color intensities and numbers of these exposed axons, which confirmed that the loss of the atg13 gene indeed impaired myelin integrity in muscle-serving nerve fibers.

### Genetic depletion of ATG13 exacerbates deficits in energy metabolism and muscle fatigue following treadmill exercise

Next, we examined whether a stressor increases glycolytic lactic acid production (ECAR glycolytic capacity) and mitochondrial ATP production deficiency (OCR reserve capacity) in Tg^+/−ATG13^ mice. To induce stress, Tg^+/−ATG13^ mice were subjected to 15 min of treadmill exercise at 14 rpm and rested for 4 h, after which their splenic Mφs were isolated to measure the mitochondrial OCR and glycolytic ECAR, as described in the methods section. First, the mitochondrial OCR (Fig. 9A) did not significantly decrease basal respiration, ATP-linked respiration or the ATP turnover rate or reserve capacity in NTg mice before or after treadmill exercise. Interestingly, after treadmill exercise, the OCR assay (Fig. 9B) revealed significant decreases in basal respiration, ATP utilization and spare or reserve capacity. Quantitative assessment of reserve capacity, an indicator of OXPHOS activity under stress conditions, revealed significant impairment (Fig. 9C) in the splenic macrophages of Tg^+/−ATG13^ mice 4 h after treadmill exercise. Next, we performed an ECAR assay to evaluate glycolysis., the ECAR (Fig. 9D**)** revealed no change in glycolytic capacity under either basal or stressed conditions in NTg mice before or after exercise. However, there was a modest induction in the spare glycolytic capacity of splenic Mφs from Tg^+/−ATG13^ mice (*n* = 3) 4 h after exercise, as shown by the last phase (post-2DG) of the ECAR curve (Fig. 9E) followed by a histogram analysis (Fig. 9F). To corroborate our findings in ECAR assay, LDH assay (Fig. 9G) revealed that Tg Mφs were associated with increased LDH under basal condition, which exacerbated at 4 h post-treadmill condition, whereas no change in LDH release was observed in NTg Mφs before and after treadmill conditions. Taken together, these results indicate that atg13 depletion may exacerbate the reduction in mitochondrial ATP production, increase in glycolytic capacity, and elevated LDH release in Tg^+/−ATG13^ mice following treadmill exercise.

Next, we investigated whether post-treadmill exacerbation of mitochondrial and glycolytic impairments in energy metabolism contributed to muscle fatigue in Tg^+/−ATG13^ mice. The open-field behavioral studies (Fig. 10A) indicated that Tg^+/−ATG13^ mice but not NTg mice had severe movement deficits that worsened even after 2 days post-Treadmill exercise. Quantitative estimations of average speed (Fig. 10B) and mobility time (Fig. 10C) further confirmed that treadmill exercise indeed worsened the mobility of Tg^+/−ATG13^ mice but not of NTg mice 2 days after treadmill exercise. To understand muscle weakness at the molecular level, we next performed surface electromyography (EMG) recordings of the biceps muscle of NTg and Tg^+/−ATG13^ mice before and 2 days after treadmill exercise, followed by heatmap analysis (Fig. 10D) to determine the sum of muscle wave frequencies. The summation of muscle waves was displayed on a Hz scale for 1 min. Interestingly, the EMG recording data from Tg^+/−ATG13^ mice showed a severe decrease in muscle frequency at the basal level that worsened 2 days after treadmill exercise, whereas no notable deficit was observed in the NTg mice. To further corroborate the role of ATG13 in worsening post-treadmill muscle weakness, we performed grip strength analyses in 10–12 weeks old male NTg and Tg^+/−ATG13^ mice (*N* = 6). Mice were subjected to a grip strength apparatus before and 1 day after tread mill exercise (14 rpm /15 mins). We observed a significant (Unpaired t-test; t = 38.20, df = 600; ****p < 0.00001) muscle weakness under basal condition (Fig. 10E), which was not alleviated(Unpaired t-test; t = 25.70, df = 600; ***p < 0.0001) one day after treadmill exercise (Fig. 10F) .

Overall, our results indicate that the depletion of atg13 followed by autophagy impairment significantly affects cellular redox metabolism through disruptions in cellular and mitochondrial energy metabolism, leading to inflammation and the polarization of splenic macrophages toward the M1 phenotype. Consequently, there is strong perivascular infiltration of these inflammatory cells that results in a demyelinating response in muscle-serving nerve fibers.

## Discussion

### The genetic depletion of ATG13 may play a crucial role in the polarization of M1Mφ in spleen

 ME/CFS [38, 39] is a chronic inflammatory disease [40] characterized by severe muscle fatigue [41] that worsens after physical or mental exhaustion [42]. However, the underlying molecular mechanism is still unknown. Our previous study indicated that disruption of autophagy due to LacZ-mediated suppression of the *atg13* gene induces severe muscle weakness, which is exacerbated after treadmill exercise. This observation intrigued us to study the underlying molecular mechanism in detail. To determine the molecular role of the atg13 gene, we generated universal cre-loxP conditional atg13 KO mice. Although homozygous KO mice are not viable, hemizygous KO mice (Tg^+/−ATG13^) are viable and exhibit a strong reduction in atg13 gene expression in the spleen. Interestingly, these Tg^+/−ATG13^ mice had significantly enlarged and inflamed spleens with an increased number of Mφs in the red pulp, suggesting the potential role of Mφs in systemic inflammation. The red pulp of the spleen is composed of sinusoids, which are open capillaries containing monocytes and macrophages. Resident Mφs in red pulp play crucial physiological roles in regulating antigenic responses and removing aged erythrocytes. Our histochemical studies indicated that genetic ablation of the atg13 gene increased the expression of IBA-1 in these Mφs, suggesting a direct role of *atg13* ablation in inducing an inflammatory phenotype in these cells. Further characterization of different surface proteins by flow cytometry confirmed that these activated Mφs acquired the M1 inflammatory phenotype and strongly expressed M1 surface proteins, including CD40 and CD86. Similar flow cytometry analyses revealed that the acquisition of M1 properties also accompanies the downregulation of M2 surface proteins such as CD163 and CD206. While characterizing the functional properties of these Mφs, our analyses revealed that these cells were strongly associated with iNOS and activated NF-κB, confirming the inflammatory M1 phenotype of these cells. Additionally, these cells lost phagocytic activity, exhibited decreased lysosomal function, and exhibited downregulated arginase expression, indicating that the loss of M2Mφ function was due to atg13 gene ablation. Consistent with our current findings, our study and other studies revealed that the activation of NFκB, dysregulation of NO production, and redox imbalance could play critical roles in the pathogenesis of ME/CFS. In ME/CFS, M1 macrophages, which are proinflammatory, are hypothesized to be relevant to the disease process. A study [43] suggested that classical monocytes in ME/CFS patients tend to migrate to tissues and become macrophages. A high-resolution transcriptomics study after analyzing 13,000 transcriptomes from 33 ME/CFS patients followed by functional network analysis also revealed the upregulation of inflammatory mediators, such as IL-8, NFκB, and TNF-α, associated with M1Mφ function. Our previous study also demonstrated the activation of STAT3 in these inflammatory Mφs, and the downstream expression of inflammatory cytokines such as IL6 and RANTES also plays a key role in ME/CFS.

### Mitochondrial deficit of energy metabolism, redox imbalance and elevated ROS production in Tg+/- ATG13 mice

 We studied the molecular mechanism through which atg13 gene ablation stimulates polarization toward the M1 phenotype. The depletion of the atg13 gene caused an increase in the production of ROS, suggesting that there may be dysregulation of cellular energy metabolism due to disrupted autophagy. Seahorse OCR analysis revealed that atg13 depletion significantly compromised basal and stress-driven mitochondrial oxidative phosphorylation (OXPHOS) in Tg^+/−ATG13^ Mφs. Interestingly, the mitochondrial deficiency in OXPHOS worsened after treadmill exercise. On the other hand, the ECAR analysis indicated that atg13 inhibition enhanced the glycolytic flux, which was augmented after treadmill exercise suggesting that the sustenance of glycolysis is required for the maintenance of the M1 phenotype. Given that the extracellular acidification rate (ECAR) is a real-time indicator of overall lactate production [44], the absence of ATG13 may increase lactate production following stress. We indeed observed that Tg Mφs were associated with elevated LDH release and lactate production under both basal and PTM conditions. Elevated levels of inactivated phospho-ATG13 have been previously reported [20] in the plasma samples of ME/CFS patients, indicating that the current findings are highly pertinent to the pathogenesis of ME/CFS. Moreover, the study of mitochondrial OXPHOS showed that ME/CFS patients have a deficiency in energy metabolism in immune cells, highlighting the relevance of our current research to the molecular mechanisms of disease progression.

Mitochondrial deficiency in energy metabolism, redox imbalance, and augmented glycolytic capacity contribute to our understanding of the polarization of Mφ cells to the M1 phenotype. However, the mechanism by which these metabolic deficits trigger an inflammatory response is not yet understood. Our current findings suggest that increased nitrosative stress diminishes the deacetylase enzymatic activity of Sirtuin 1 (SIRT1), causing upregulation of the acetylated fraction of activated NFκB. In contrast, SIRT2 activity was unaltered in the spleen, suggesting that diminished SIRT1 activity may be responsible for stimulating the inflammation of splenic Mφs.

Our current manuscript also highlights the potential mechanism of inflammation in skeletal muscle tissue through the augmented infiltration of these M1Mφ cells in the perivascular network of the muscle parenchyma. Subsequent histological analysis revealed significant impairment of myelin integrity in the muscle-serving nerve fibers of nerve spindles. EMG data also indicated that the decrease in skeletal muscle strength in these Tg^+/−ATG13^ mice was exacerbated following treadmill exercise.

In summary, our research highlights a novel mechanism of inflammation caused by depletion of the atg13 gene followed by autophagy impairment, which combines mitochondrial impairment of energy metabolism, increased ROS production, upregulation of inflammatory markers, polarization of M1Mφs, enhanced infiltration into the muscle vasculature, impaired myelin integrity, and ultimately chronic muscle weakness.

### Future directions and limitations

We are currently conducting a decentralized observational clinical trial of the mTOR inhibitor rapamycin for the alleviation of clinical symptoms of fatigue in mothers and CFSs. We collected plasma and PBMCs from the participating subjects before and at 30, 60, and 90 days after administration of 6 mg/week rapamycin. In the future, we will conduct an M1/M2 polarization study of PBMC-derived Mφs. Additionally, we investigated whether plasma-borne factors could polarize human Mφs to the M1 phenotype. Another future direction of our current study is to understand the role of autophagy and the mitochondrial impairment of energy metabolism in maintaining the viability and inflammatory properties of different types of T lymphocytes that will further highlight the role of ATG13 in systemic inflammation. One limitation of our current study is the gender biasness. Since the universal cre mice is applicable to the genetic deletion of *atg13* gene only in male mice, our study primarily focuses the pathological changes in males. Another limitation of this study is using the self-adhesion method [45] to purify Mφs, as other antigen-presenting cells may still be present. While magnetic bead-based separation could address this issue, it often reduces cell viability, alters surface properties, and affects self-propagation in culture.

## Supplementary Information

Below is the link to the electronic supplementary material.

Supplementary Material 1

## Data Availability

The electronic datasheet associated with this paper can be available in Mendeley database using following link: https://data.mendeley.com/datasets/w8kvmbmh4p/1 and DOI: 10.17632/w8kvmbmh4p.1.

## Abbreviations

ATG13: Autophagy-Related Protein 13
Mφ: Macrophage
Tg^+/−ATG13^: Hemizygous Deletion of ATG13 Transgenic Mouse
CD: Cluster of Differentiation
ME/CFS: Myalgic Encephalomyelitis/Chronic Fatigue Syndrome
PEM: Postexertional Malaise
ROS: Reactive Oxygen Species
IF: Immunofluorescence
IB: Immunoblot
OCR: Oxygen Consumption Rate,
FACS: Flow Cytometry-Activated Cell Sorting
ECAR: Extracellular Acidification Rate
OXPHOS: Oxidative Phosphorylation
