# Supplementary figures and images for: Genetic depletion of the early autophagy protein ATG13 impairs mitochondrial energy metabolism, augments oxidative stress, induces the polarization of macrophages to the M1 inflammatory mode, and compromises myelin integrity in skeletal muscle
Source: Inflamm Res. 2026 Jan 27;75(1):26. doi: 10.1007/s00011-025-02158-6 (PMC12847126; doi:10.1007/s00011-025-02158-6)

**Fig. 1E**

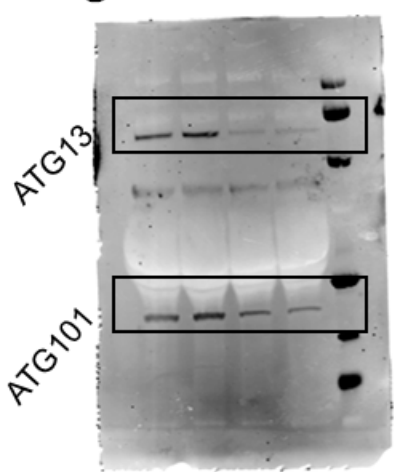

**Fig. 2E**

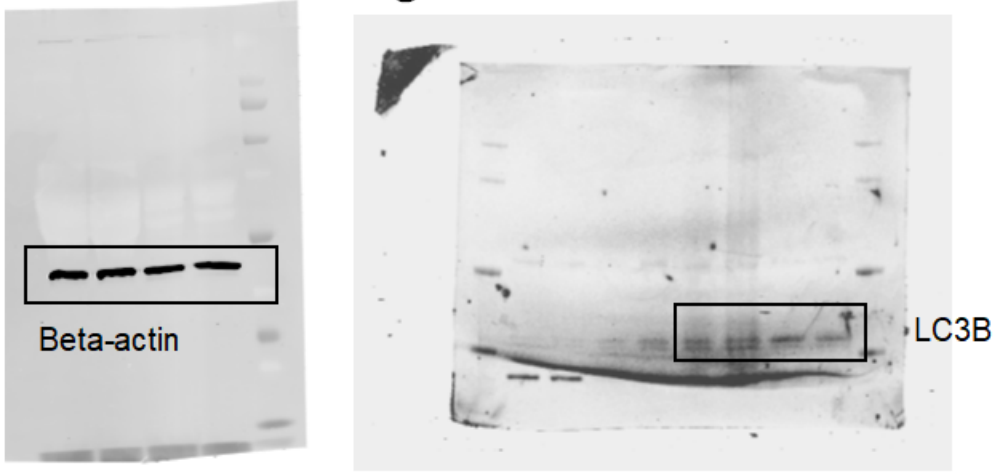

**Fig. 3C**

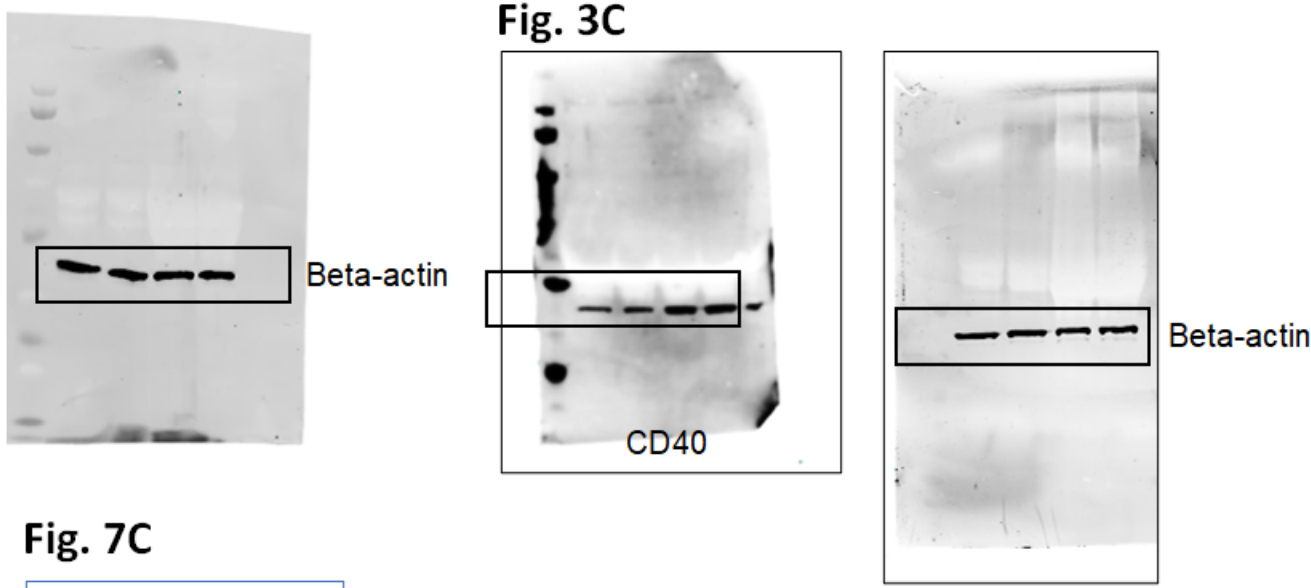

**Fig. 7C**

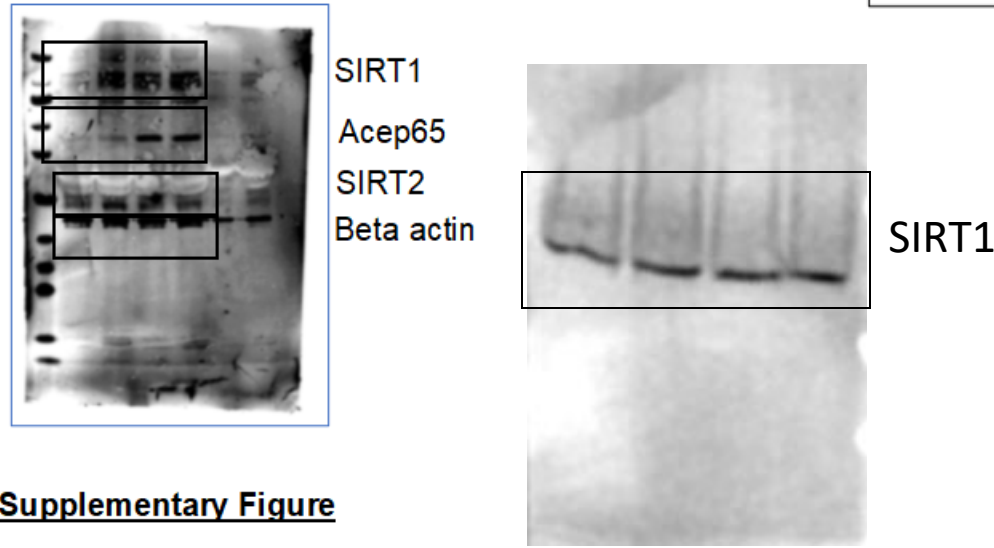

**Supplementary Figure**

Supplement: Supplementary file 1 — Supplementary Material 1 [file 11_2025_2158_MOESM1_ESM.pdf]
